# ClusterDE: a post-clustering differential expression (DE) method robust to false-positive inflation caused by double dipping

**DOI:** 10.21203/rs.3.rs-3211191/v1

**Published:** 2023-08-02

**Authors:** Dongyuan Song, Kexin Li, Xinzhou Ge, Jingyi Jessica Li

**Affiliations:** 1 Bioinformatics Interdepartmental Ph.D. Program, University of California, Los Angeles, CA 90095-7246; 2 Department of Statistics, University of California, Los Angeles, CA 90095-1554; 3 Department of Human Genetics, University of California, Los Angeles, CA 90095-7088; 4 Department of Computational Medicine, University of California, Los Angeles, CA 90095-1766; 5 Department of Biostatistics, University of California, Los Angeles, CA 90095-1772; 6 Radcliffe Institute for Advanced Study, Harvard University, Cambridge, MA 02138

## Abstract

In typical single-cell RNA-seq (scRNA-seq) data analysis, a clustering algorithm is applied to find putative cell types as clusters, and then a statistical differential expression (DE) test is employed to identify the differentially expressed (DE) genes between the cell clusters. However, this common procedure uses the same data twice, an issue known as “double dipping”: the same data is used twice to define cell clusters as potential cell types and DE genes as potential cell-type marker genes, leading to false-positive cell-type marker genes even when the cell clusters are spurious. To overcome this challenge, we propose ClusterDE, a post-clustering DE method for controlling the false discovery rate (FDR) of identified DE genes regardless of clustering quality, which can work as an add-on to popular pipelines such as Seurat. The core idea of ClusterDE is to generate real-data-based synthetic null data containing only one cluster, as contrast to the real data, for evaluating the whole procedure of clustering followed by a DE test. Using comprehensive simulation and real data analysis, we show that ClusterDE has not only solid FDR control but also the ability to identify cell-type marker genes as top DE genes and distinguish them from housekeeping genes. ClusterDE is fast, transparent, and adaptive to a wide range of clustering algorithms and DE tests. Besides scRNA-seq data, ClusterDE is generally applicable to post-clustering DE analysis, including single-cell multi-omics data analysis.

## Introduction

The recent development of single-cell RNA-seq (scRNA-seq) technologies has revolutionized transcriptomic studies by providing unprecedented pictures of gene expression within individual cells. A major task of scRNA-seq data analysis is to annotate cell types and understand their biological differences. Hence, the standard workflow of analyzing scRNA-seq data includes two steps: (1) clustering cells to find potential cell types, and (2) finding differentially expressed (DE) genes between cell clusters as potential cell-type marker genes [1, 2].

Although this post-clustering differential expression (DE) procedure is used in the state-of-the-art scRNA-seq analysis pipelines such as the R package Seurat (ref [3]) and the Python module Scanpy (ref [4]), researchers have realized that this procedure is conceptually problematic. For instance, Seurat contains the warning message that “*P* values should be interpreted cautiously, as the genes used for clustering are the same genes tested for differential expression.” This issue is commonly referred to as “double dipping,” meaning that the same gene expression data are used twice to define cell clusters and DE genes, thus leading to an inflated false discovery rate (FDR) in identifying post-clustering DE genes as putative cell-type marker genes when the cell clusters are spurious.

**Figure 1:**
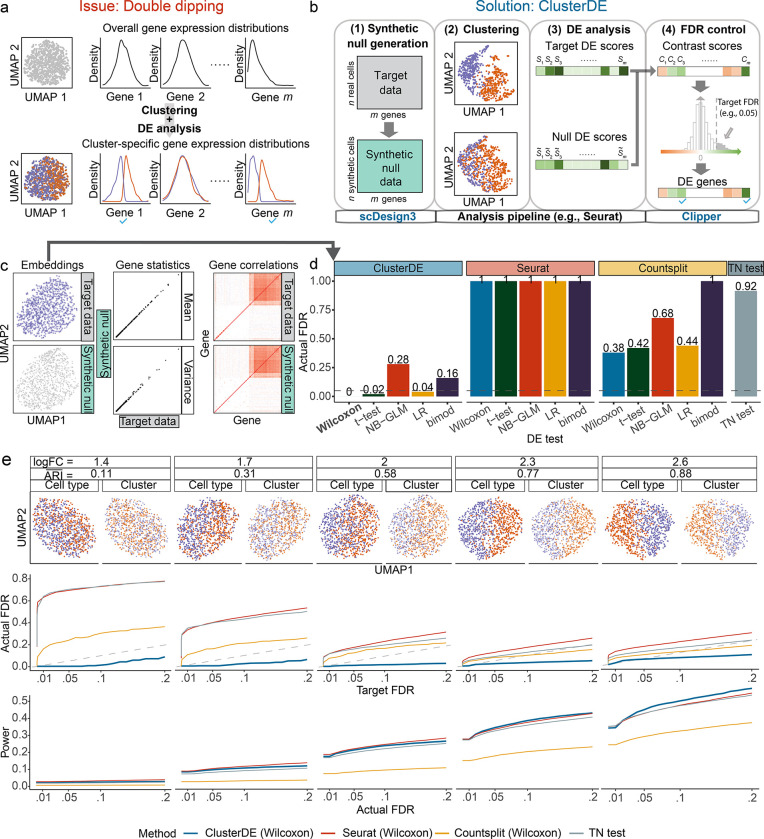
ClusterDE is a solution to the double-dipping issue in post-clustering DE analysis. **a**, An illustration of the double-dipping issue. Each gene’s expression follows a unimodal distribution when cells come from a homogeneous cell type. However, if clustering divides the cells into two clusters, certain genes are “forced” to have different distributions between the two clusters. **b**, An overview of the ClusterDE test. Given the “target data” (real data), ClusterDE employs the simulator scDesign3 (ref [18]) to generate the corresponding “synthetic null data,” which contains synthetic cells from one “hypothetical” cell type (the null hypothesis) to mimic the real cells but fill any gap between real cell types if existent. Then ClusterDE applies a clustering algorithm followed by a DE test to both the target data and the synthetic null data in parallel, yielding two DE scores for each gene (a “target DE score” and a “null DE score”). Finally, ClusterDE uses the FDR-control method Clipper (ref [19]) to calculate a contrast score based on the two DE scores for each gene. ClusterDE identifies DE genes as those whose contrast scores exceed the threshold, which is determined by finding a contrast score threshold (represented by the vertical dashed line) based on the contrast score distribution and the desired target FDR (e.g., 0.05). **c**, When the target data contained cells from a single type (simulation; see Methods “Simulation design with one cell type and zero DE genes”), the synthetic null data generated by ClusterDE resembled the target data well in terms of UMAP cell embeddings (left), per-gene expression mean and variance statistics (middle), and gene-gene correlations (right). **d**, On the target data in c, ClusterDE (with five DE tests) outperformed existing methods—including Seurat (which does not consider double dipping), Countsplit (which aims to address double dipping and works with any DE test), and TN test (which aims to address double dipping and has its own DE test)—in FDR control. The horizontal dashed line indicates the target FDR of 0.05. The five DE tests are the Wilcoxon rank sum test (Wilcoxon), t-test, negative binomial generalized linear model (NB-GLM), logistic regression model predicting cluster membership with likelihood-ratio test (LR), and likelihood-ratio test for single cell gene expression (bimod). **e**, The FDRs and power of ClusterDE and the existing methods under various severity levels of double dipping. The log fold change (logFC) summarizes the average gene expression difference between two cell types in simulation (see Methods “Simulation design with two cell types and 200 DE genes”). Corresponding to a small logFC, a small adjusted Rand index (ARI) represents a bad agreement between cell clusters and cell types, representing a more severe double-dipping issue. Across various severity levels of double dipping, ClusterDE controlled the FDRs under the target FDR thresholds (diagonal dashed line) and achieved comparable or higher power compared to the existing methods at the same actual FDRs.

We illustrate the double-dipping issue in Fig. 1a, a scenario where only a single cell type exists, and no genes should be identified as between-cell-type DE genes. However, as clustering is based on gene expression data, certain genes would be correlated with the resulting cell clusters if their expression patterns drive the clustering. Hence, these genes would have different conditional distributions in the two cell clusters and subsequently be identified as between-cell-cluster DE genes, but they are false-positive between-cell-type DE genes. Therefore, this double-dipping issue would inflate the false discovery rate (FDR), the expected proportion of false-positive between-cell-type DE genes among all identified DE genes.

Two attempts to solve the double-dipping issue include the truncated normal (TN) test and the Countsplit method. The first method TN test has five steps: (1) splitting cells into two sets: training cells and test cells [5]; (2) applying a clustering algorithm to the training cells to find two clusters; (3) training a support vector machine classifier on the training cells to predict a cell’s cluster label from the cell’s gene expression vector; (4) using the trained classifier to predict the test cells’ cluster labels; (5) finding DE genes between the two test cell clusters using the TN test. Instead of splitting cells, the second method Countsplit splits the scRNA-seq count matrix into two count matrices of the same dimensions (cells and genes)—a training matrix and a test matrix—by a procedure called data thinning [6]. Since the two matrices have exactly matched cells, Countsplit finds cell clusters by applying a clustering algorithm to the training matrix, and it subsequently identifies DE genes by applying a DE test to the test matrix given the cell clusters. Despite the claims made by the TN test and Countsplit that they can provide well-calibrated *P* values, uniformly distributed between 0 and 1 under the null hypotheses, our findings indicate that their *P* values are anti-conservative in the presence of gene-gene correlations (see Results). The reason behind this issue is that the validity check of *P* values in the TN test and Countsplit papers relied on simulation studies that implicitly assumed genes to be independent [5, 6], an assumption that does not hold in real scRNA-seq data. As a result, the *P* value calibration issue would lead to inflated FDRs when the TN test and Countsplit are applied to real scRNA-seq data..

In addition to the TN test and Countsplit, several cluster-free DE tests have been developed to circumvent the double-dipping issue by bypassing the cell clustering step [7–12]. However, it is important to note that these cluster-free methods do not aim to identify potential cell types. Consequently, the DE genes identified by these methods cannot be interpreted as marker genes for specific cell types, unlike the DE genes identified after clustering. In other words, the cluster-free DE genes and the post-clustering DE genes serve different purposes and are not conceptually comparable. Another stream of methods has been developed to assess the quality of clustering results, e.g., the “purity” of a cluster or if two clusters should be merged [13–17]. However, these methods do not provide a direct statistical test for identifying DE genes, and it remains difficult to determine the threshold for clustering quality above which double dipping is not a concern. In this study, we focus on addressing the inflated FDR issue when using post-clustering DE genes as cell-type marker genes. Hence, we do not consider cluster-free DE tests and clustering assessment methods as competing alternatives in our investigation.

Here we introduce ClusterDE, a post-clustering DE method for identifying potential cell-type marker genes by avoiding the inflated FDR issue due to double dipping. It is worth noting that ClusterDE is not designed to replace any existing pipelines for clustering followed by DE analysis (e.g., Seurat); instead, ClusterDE works simply as an add-on to an existing pipeline for achieving more reliable discoveries. In particular, ClusterDE controls the FDR for identifying cell-type marker genes even when the cell clusters are spurious. As an efficient and interpretable method, ClusterDE adapts to the most widely used pipelines Seurat (ref [3]) and Scanpy (ref [4]), which include a wide range of clustering algorithms and DE tests. We benchmarked ClusterDE against the default Seurat (which includes double dipping), the TN test, and Countsplit, each of which includes a cell clustering step and a DE analysis step. Specifically, to align with the prevailing practices in single-cell data analysis, we employed the default Seurat clustering algorithm (which involves data processing steps followed by the Louvain algorithm) for cell clustering; for DE analysis, we evaluated five widely used DE tests (e.g., the Wilcoxon rank-sum test and the two-sample *t* test) included in the Seurat package, with the exception of the TN test, which utilizes its own DE test. Our benchmarking results demonstrate that ClusterDE is the only method that effectively controls the FDR across varying thresholds. Moreover, ClusterDE achieves comparable or superior statistical power compared to the other three methods. When applied to the scRNA-seq data of five homogeneous cell lines, ClusterDE successfully avoids finding false-positive DE genes. In contrast, Seurat, the TN test, and Countsplit yield thousands of DE genes due to double dipping. Moreover, when applied to a well-studied peripheral blood mononuclear cells (PBMC) scRNA-seq dataset with two biological replicates and four protocols, ClusterDE excels at discovering the cell-type marker genes of CD14^+^ monocytes and CD16^+^ monocytes as its top DE genes, while Seurat’s top DE genes contain many housekeeping genes. Besides the ability to control the FDR and identify cell-type marker genes, ClusterDE has a notable practical advantage for allowing users to dissect an abstract statistical null hypothesis as concrete synthetic null data, so users can decide whether the synthetic null data accurately reflects the negative control scenario they have in mind, and if not, how the synthetic null generation should be adjusted.

## Results

### ClusterDE uses a contrastive strategy to identify reliable DE genes robust to double dipping

The ClusterDE test consists of four major steps (Fig. 1b), with its core idea being to establish a negative control for the entire computational pipeline that includes cell clustering followed by DE analysis. This contrastive strategy enables the identification of trustworthy DE genes by comparing the result from real-data analysis with that from the negative-control analysis. To implement this strategy, we introduce a null model that assumes the cells of interest (i.e., the cells divided into two clusters and subject to DE analysis, referred to as the “target data”) are from a homogeneous cell type, where no between-cell-type DE genes should be detected.

In step 1 of ClusterDE, we use the model-based simulator scDesign3 (ref [18]) to generate “synthetic null data” that mimic the target data but represent a homogeneous cell type, with the same number of cells and the same genes as in the target data. Fig. S1 illustrates the synthetic null generation process, with the mathematical details described in Methods. Fig. 1c and Fig. S2 show that the synthetic null data preserve the per-gene mean and variance statistics, as well as the gene-gene correlations in the target data. Meanwhile, irrespective of the clustering pattern in the target data, the synthetic null data exhibit a homogeneous cell cluster, which is specified as the “null model” for a single cell type in scDesign3.

In steps 2 and 3 of ClusterDE, users have the flexibility to specify a clustering algorithm and a DE test, respectively, to analyze the target data and the synthetic null data in parallel. For example, users may use the Seurat pipeline for clustering and DE analysis. These two steps yield a “target DE score” and a “null DE score” for each gene. Specifically, we define a gene’s DE score as a summary statistic measuring the difference of the gene’s expression values in two clusters; a higher DE score indicates that the gene is more likely DE. For example, the DE score is by default defined as the negative logarithm of the *P* value obtained from a statistical DE test (e.g., the Wilcoxon rank-sum test).

Finally, in step 4 of ClusterDE, a “contrast core” is computed for each gene by subtracting the gene’s null DE score from its target DE score. True non-DE genes are expected to have contrast scores symmetrically distributed around 0. Then ClusterDE uses the FDR control method Clipper (ref [19]) to determine a contrast score cutoff based on a target FDR (e.g., 0.05). Genes with contrast scores greater than or equal to the cutoff are identified as DE genes.

The detailed procedure of ClusterDE is described in Methods.

### ClusterDE achieves reliable FDR control and good statistical power under double dipping

We conducted extensive simulation studies to validate ClusterDE as a post-clustering DE method with reliable FDR control under double dipping. We also compared ClusterDE with Seurat, the most widely used analysis pipeline that involves double dipping, and two existing methods that attempted to address the double-dipping issue—the TN test (ref [5]) and Countsplit (ref [6]). In the cell clustering step of all four methods, we used the default Seurat clustering as in most scRNA-seq data analyses. In the DE analysis step of ClusterDE, Seurat, and Countsplit, we considered five DE tests in the Seurat package: the Wilcoxon rank-sum test (Wilcoxon; the default option in the Seurat package), the two-sample *t* test (t-test), the negative binomial generalized linear model (NB-GLM), the logistic regression (LR), and the likelihood-ratio test (bimod). The TN test is an exception because it uses its own TN test in the DE analysis step. As Seurat, Countsplit, and the TN test all output a *P* value for each gene, we applied the Benjamini-Hochberg (BH) procedure to all genes’ *P* values to find a *P* value cutoff given a target FDR (e.g., 0.05). Genes with *P* values less than or equal to the cutoff are identified as DE genes.

In the first simulation setting, which represents the most severe double-dipping scenario, we simulated the target data from a single cell type by mimicking the naïve cytotoxic T cells in a real dataset (ref [20]) (Fig. 1c top left; see Methods section “Simulation design”), where any identified DE genes should be considered false discoveries. At the target FDR of 0.05, all three existing methods—Seurat, Countsplit, and the TN test—were unable to control the actual FDR under 0.05 (Fig. 1d). As expected, the double-dipping approach employed by Seurat exhibited the worst performance, with all five DE tests yielding actual FDRs of 1. Although Countsplit and the TN test were designed to overcome the FDR inflation issue caused by double dipping, their actual FDRs still far exceeded 0.05. The reason is that their *P* values are anti-conservative in the presence of gene-gene correlations (Fig. S3 right), although their own simulation studies verified their *P*-value validity under unrealistic settings where genes are assumed to be independent [5, 6]. In contrast, ClusterDE successfully controlled the FDRs under 0.05 for three out of the five DE tests: Wilcoxon, t-test, and LR (Fig. 1d). We verified that the contrast scores calculated in step 4 of ClusterDE satisfied the symmetry requirement around zero (Fig. S3 left). Although ClusterDE did not control the actual FDRs of the NB-GLM and bimod tests under 0.05 due to possible violations of these two tests’ parametric modeling assumptions on this dataset, the FDR inflation of ClusterDE for these two tests was much less severe than that of Countsplit (ClusterDE’s actual FDRs 0.28 and 0.16 vs. Countsplit’s actual FDRs 0.68 and 1 for NB-GLM and bimod, respectively) (Fig. 1d).

In the second simulation setting, we generated datasets with varying degrees of double dipping, still by mimicking the nïve cytoxic T cells in a real dataset (ref [20]) (Fig. 1e top; see Methods section “Simulation design”). Each dataset consists of two synthetic cell types with pre-specified 200 true DE genes with varying expression level differences between the cell types, and the overall difference is summarized as the log fold change (logFC). A larger logFC indicates a greater distinction between the two cell types. After the default Seurat clustering algorithm is applied to each dataset to identify two cell clusters, the agreement between the cell clusters and the cell types is measured by the adjusted Rand index (ARI). A smaller ARI represents a more severe degree of double dipping, as illustrated by the UMAP visualizations (Fig. 1e top row). Since Wilcoxon is the default DE test in Seurat and yielded the best FDR control for both ClusterDE and Countsplit, we used Wilcoxon as the DE test in ClusterDE, Seurat, and Countsplit, while the TN test uses its own DE test. The results in Fig. 1e show that ClusterDE consistently controlled the actual FDRs across a range of target FDR thresholds under varying degrees of double dipping. In contrast, Seurat, Countsplit, and the TN test failed to control the actual FDRs under the target thresholds, and as expected, exhibited greater FDR inflation when the degree of double dipping is more severe (Fig. 1e middle row). Notably, ClusterDE achieved comparable or superior statistical power to Seurat, Countsplit, and the TN test at the same actual FDR levels (Fig. 1e bottom). These conclusions remained to hold when ClusterDE, Seurat, and Countsplit were used with the other four DE tests (t-test, NB-GLM, LR, and bimod) in the DE analysis step (Fig. S4). Moreover, to reflect the fact that cell types mostly have unbalanced cell numbers in real data, we further simulated target data in which the two synthetic cell types have size ratios of 1 : 4 and 1 : 9. In these two unbalanced scenarios, we still found ClusterDE to outperform the other three methods in terms of FDR control across target FDR thresholds and under varying degrees of double dipping. In particular, ClusterDE consistently exhibited solid FDR control and comparable or superior statistical power to the other three methods when used with Wilcoxon as the DE test (Fig. S5–Fig. S6).

Technically, ClusterDE shares with the knockoffs methods the concept of controlling the FDR by generating *in silico* negative control data [21]. The knockoffs methods are a suite of statistical methods developed for identifying important features in a high-dimensional predictive model, a supervised-learning setting different from our one-test-per-gene test setting. Roughly, the knockoffs methods generate knockoff data from real data in such a way that each feature is no longer correlated with the outcome variable given the other features, while the feature-feature correlations are preserved in the knockoff data. We applied the default model-X knockoffs method (ref [22]) to the simulated datasets—treating genes as features and the cell cluster label as the outcome variable; the results indicate that, although this method controlled the FDR, it always led to zero statistical power, making it impractical for DE gene identification. Moreover, we used the model-X knockoffs method and permutations (where each gene is independently permuted across all cells) as two alternative strategies to scDesign3 for the synthetic null generation in step 1 of ClusterDE, followed by steps 2–4 of ClusterDE. Fig. S7 shows a comparison of the target data with the synthetic null data generated by each of the three strategies. Compared with the target data, the synthetic null data generated by scDesign3 preserved per-gene mean and variance statistics and gene-gene correlations. In contrast, the synthetic null data generated by the model-X knockoffs method did not preserve gene mean and variance statistics, and the synthetic null data generated by permutations did not preserve gene-gene correlations. Hence, only the synthetic null cells generated by scDesign3 preserved the 2D UMAP cell embedding topology of the target cells except for filling the gap, if existent, between the target cell types. Our results on the simulated datasets demonstrate that scDesign3 led to the most solid FDR control and the best statistical power among the three strategies for synthetic null generation (Fig. S8).

To address the practical concern about the randomness involved in generating synthetic null data (a random sampling process from the null model fitted on target data), we conducted an analysis to assess the robustness of DE genes identified by ClusterDE. The results show that the DE genes identified by ClusterDE remain relatively stable and robust to the randomness (Fig. S9).

In summary, the above simulation studies confirm that ClusterDE is a flexible and stable method that effectively controls the FDR under varying degrees of double dipping while maintaining good statistical power.

### ClusterDE identifies cell-type marker genes and excludes housekeeping genes from its top DE genes

We applied ClusterDE to multiple real scRNA-seq datasets to demonstrate how it can enhance the rigor and biological relevance of findings from the post-clustering DE analysis. The following real data applications showcase the effectiveness of ClusterDE in identifying meaningful DE genes and improving the reliability of DE gene identification.

**Figure 2:**
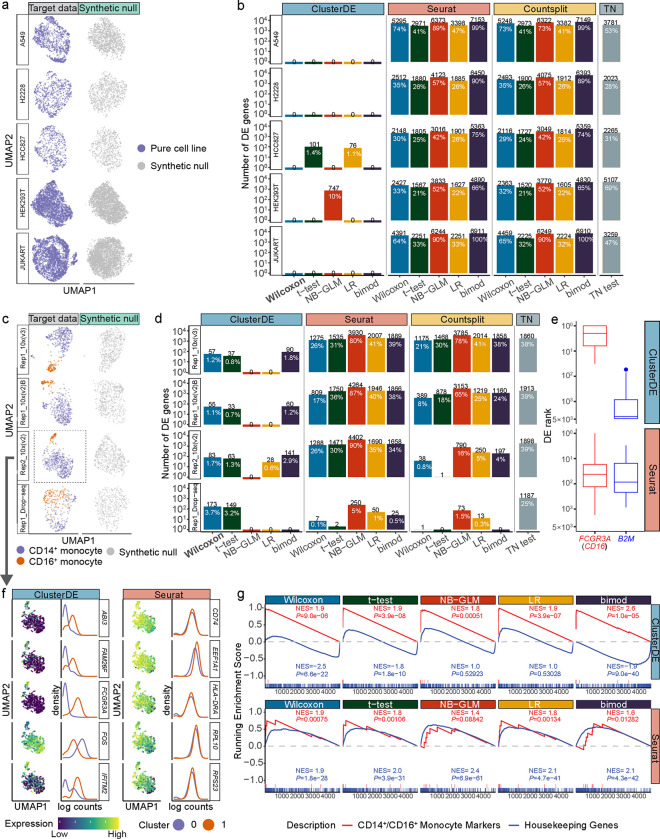
ClusterDE achieves reliable FDR control and good statistical power in identifying DE genes from real scRNA-seq data. **a**, UMAP visualizations of target data (left) and synthetic null data (right) of five cell lines. **b**, Numbers of DE genes (at the target FDR of 0.05) identified by ClusterDE and the existing methods. While all existing methods found numerous “false” DE genes within a single cell line, ClusterDE made no false discoveries when used with most DE tests. The numbers in black and white indicate the number of DE genes and the proportions of DE genes among all genes, respectively. The five DE tests are the Wilcoxon rank sum test (Wilcoxon), t-test, negative binomial generalized linear model (NB-GLM), logistic regression model predicting cluster membership with likelihood-ratio test (LR), and likelihood-ratio test for single cell gene expression (bimod). **c**, UMAP visualizations of target data (left) and synthetic null data (right) for four datasets containing two monocyte subtypes: CD14^+^ monocytes and CD16^+^ monocytes. The synthetic null data captured the global topology of the real cells in the target data while filling the gap between the two cell subtypes. The grey dashed box labels the dataset used in f and g. **d**, ClusterDE identified DE genes between the two cell subtypes. The numbers in black and white indicate the number of DE genes and the proportions of DE genes among all genes, respectively. **e**, The ranks of two exemplary genes (a monocyte subtype marker *FCGR3A* in red and a well-known housekeeping gene *B2M* in blue) in the DE gene lists of ClusterDE and Seurat across the five DE tests and the four datasets in c. In each boxplot representing the distribution of 20 ranks, the center horizontal line represents the median, and the box limits represent the upper and lower quartiles. **f**, The top DE genes identified by ClusterDE exhibited distinct expression patterns in the two cell clusters identified by Seurat clustering, a phenomenon not observed for the top DE genes identified by Seurat. For ClusterDE and Seurat, the top DE genes are defined as the common DE genes found by the five DE tests in d at the target FDR of 0.05. The UMAP plots show each top DE gene’s normalized expression levels in the dataset “Rep2_10x(V2)” (marked by the dashed box in c; see Methods section “Dimensionality reduction and visualization”). The density plots depict each top DE gene’s normalized expression distributions in the two cell clusters. **g**, Gene set enrichment analysis (GSEA) of the ranked DE gene lists identified by ClusterDE and Seurat with five DE tests from the dataset Rep2_10x(V2). The red lines represent the enrichment of the CD14^+^/CD16^+^ monocyte marker gene set, and the blue lines represent the enrichment of the housekeeping gene set. The normalized enrichment score (NES) reflects the direction and magnitude of enrichment, and the *P* value indicates the significance of enrichment.

In the first application, we collected five datasets of pure cell lines [23, 24], so the cells in each dataset can be trusted as a homogeneous population that should not be divided into more than one cluster (Fig. 2a left). Hence, any post-clustering DE genes identified from these datasets should not be interpreted as between-cell-type DE genes. We used these five datasets as real-data negative examples to demonstrate the inflated FDRs of existing methods and the effectiveness of ClusterDE in removing the FDR inflation. As a sanity check of ClusterDE, we first verified that the synthetic null data resembled the target data (Fig. 2a right). Applying ClusterDE, Seurat, Countsplit, and the TN test to the five datasets, we found that all methods except ClusterDE identified thousands of DE genes, in many cases even more than 50% of all genes, indicating severely inflated false discoveries at the target FDR of 5%. In contrast, ClusterDE found zero DE genes in 22 out of 25 cases when used with the five DE tests (Wilcoxon, t-test, NB-GLM, LR, and bimod) on the five datasets. In particular, ClusterDE with Wilcoxon consistently found zero DE genes from the five datasets. Hence, we set Wilcoxon as the default DE test in ClusterDE.

In the second application, we collected eight PBMC datasets of CD14^+^/CD16^+^ monocytes (ref [25]) to demonstrate that ClusterDE can effectively detect known or potential marker genes of the two cell subtypes. The eight datasets were generated from two technical replicates by four unique molecular identifier (UMI) based scRNA-seq protocols (10X Genomics Versions 2 and 3, Drop-seq, and inDrop). After applying the default Seurat clustering to identify two clusters in each of the eight datasets, we found four datasets to have relatively accurate clustering results (ARI > 0.5; Fig. 2c left, Fig. S10 top), while the other four datasets had clusters poorly matched with the two monocyte subtypes (ARI < 0.2; Fig. S10 bottom). Hence, we expected that an effective post-clustering DE method would be able to detect meaningful marker genes for monocyte subtypes in the first four datasets, but we did not expect the same level of effectiveness for the latter four datasets. Hence, we focused on the analysis results of the first four datasets. As a sanity check of ClusterDE, we first verified that the synthetic null data resembled the target data but had the gap filled between CD14^+^ monocytes and CD16^+^ monocytes, representing a single “hypothetical” cell type in each dataset (Fig. 2c right). Applied to the first four datasets with relatively accurate clustering results, ClusterDE with Wilcoxon identified 55–173 DE genes (1–4% of all genes) at the target FDR of 5%, while Seurat and Countsplit identified 1–1,288 DE genes (0–26% of all genes), and the TN test consistently identified at least 1,187 genes (25% of all genes) (Fig. 2d). Given our knowledge that the two monocyte subtypes are not drastically different, we did not expect thousands of genes to be identified as potential subtype marker genes. Hence, we deemed the number of DE genes identified by ClusterDE to be more reasonable.

Examining the post-clustering DE genes identified by ClusterDE or Seurat across the five DE tests on the four datasets (so ClusterDE and Seurat each had 20 DE gene lists), we found that ClusterDE better distinguished known subtype marker genes from housekeeping genes than Seurat did. This distinction was evident in the ranking of specific genes in the DE gene lists. For example, we considered the genes *FCGR3A* (*CD16*), a canonical marker for distinguishing CD14^+^ monocytes and CD16^+^ monocytes, and *B2M*, a widely recognized housekeeping gene expressed across various cell types [26]. Notably, ClusterDE consistently ranked *FCGR3A* among its top DE genes (with ranks approximately between 1 and 10) while placing *B2M* consistently low in its DE gene lists (with ranks below 1,000 in most cases) (Fig. 2e top). In contrast, Seurat ranked the two genes similarly (with ranks between 10 and 100) in its DE gene lists (Fig. 2e bottom), making it impossible to discern which of the two genes is more likely a subtype marker without prior knowledge.

Next, using one of the four datasets “Rep2_10x(V2)” as an example, we examined the five most frequently identified post-clustering DE genes (defined based on the top 50 DE genes identified by each of the five DE tests) by ClusterDE or Seurat (Fig. 2f). Again, the two clusters were found by the default Seurat clustering, and ClusterDE and Seurat both used these two clusters for post-clustering DE analysis. Our analysis found that the five genes identified by ClusterDE all exhibited distinct distributions of normalized expression levels between the two clusters, while the five genes identified by Seurat all had almost indistinguishable distributions between the two clusters (Fig. 2f). Further, we examined the enrichment of two gene sets—known CD14^+^/CD16^+^ monocyte markers and housekeeping genes—in the post-clustering DE gene lists outputted by ClusterDE and Seurat. The gene set enrichment analysis (GSEA) revealed that the known monocyte markers had strong enrichment in the top-ranked DE genes identified by ClusterDE, exhibiting a clear distinction from the housekeeping genes (Fig. 2g top). In contrast, the monocyte makers exhibited less enrichment in the top-ranked DE genes identified by Seurat; what is worse, they had a similar enrichment trend as the housekeeping genes, indicating that Seurat had the monocyte markers and the housekeeping genes hardly distinguishable in its ranked DE gene list (Fig. 2g bottom). The GSEA results on the other three datasets confirmed that ClusterDE better distinguished the monocyte markers from the housekeeping genes than Seurat (Fig. S12).

Considering the common analysis practice that only the top *k* DE genes (e.g., *k* = 100) are used for further investigation, we summarized the numbers of monocyte markers and housekeeping genes among the top *k* = 1 to 100 DE genes identified by ClusterDE or Seurat across the five DE tests on the four datasets. Fig. S13 shows that ClusterDE found more monocyte markers and fewer housekeeping genes among the top DE genes than Seurat. To further explain why ClusterDE can better distinguish monocyte markers and housekeeping genes, we used the minus-average (MA) plots (ref [27]) to demonstrate the effectiveness of using synthetic null as a contrast to remove housekeeping genes from the top DE genes. From the MA plots (Fig. S14), we observed that four exemplary housekeeping genes (*ACTB*, *ACTG1*, *B2M*, and *GAPDH*; marked in blue in Fig. S14) had both large target DE scores and large null DE scores, resulting in close-to-zero contrast scores, so these genes were not found by ClusterDE as top DE genes. However, these four genes were found by Seurat as top DE genes due to their large target DE scores. On the other hand, we examined four exemplary monocyte markers (*CD14*, *FCGR3A*, *MS4A7*, and *LYZ*; marked in red in Fig. S14) and found them to have large target DE scores but small null DE scores, so they were identified as top DE genes by ClusterDE.

In conclusion, ClusterDE is an effective solution to the double-dipping issue in post-clustering DE analysis. We note that ClusterDE focuses on identifying potential cell-type marker genes for cell-type annotation, so ClusterDE does not aim to capture the within-cell-type heterogeneity that reflects continuous cell state changes. Notably, ClusterDE adapts to a wide range of clustering algorithms and DE tests. Through extensive simulation studies and real data analysis, we demonstrated that ClusterDE effectively avoids false discoveries caused by double dipping and identifies biologically meaningful cell-type markers. For post-clustering DE analysis with more than two clusters, we recommend using ClusterDE in a stepwise manner, possibly following a cell cluster hierarchy constructed based on cluster similarities (Fig. S15). That is, users compare a pair of ambiguous clusters at each step, so the post-clustering DE genes can be used to decide whether the two clusters are biologically meaningful and should be distinct. Finally, while ClusterDE focuses on the double-dipping problem in the post-clustering DE analysis, the concept of synthetic null data (*in silico* negative control) can be readily extended to other analyses also affected by double dipping, such as post-pseudotime DE analysis [28] and data integration analysis. As double dipping is almost surely unavoidable in single-cell data analysis due to the lack of external knowledge, we proposed a general strategy to reduce false discoveries caused by double dipping by setting up synthetic null data and using a contrastive strategy to find more reliable discoveries.

## Online Methods

### Practical guidelines for ClusterDE usage

ClusterDE is designed to find potential cell-type marker genes via pairwise comparisons of cell clusters that might be ambiguous. In practice, we recommend using ClusterDE in the following steps.

1. Given a set of cell clusters, find two clusters that may be defined as potential cell types or subtypes. If users use Seurat, they may use the function BuildClusterTree to construct a hierarchy of the clusters and examine two leaf clusters whose distinctions are ambiguous.
2. Given the two chosen cell clusters, construct a data subset that contains only the cells in these two clusters.
3. Input the data subset as the “target data” into ClusterDE.
4. Examine the DE genes outputted by ClusterDE and decide whether the two cell clusters are biologically meaningful cell types or subtypes.

It is worth noting that ClusterDE does not provide an automatic decision about whether two clusters should be merged, unlike the methods that directly assess the quality of clusters [13–17]. Instead, ClusterDE focuses on identifying trustworthy post-clustering DE genes as potential cell-type marker genes, enabling researchers to gain biological insights into clusters by investigating the specific genes that distinguish the clusters. Hence, in contrast to the clustering quality assessment methods, ClusterDE empowers researchers to explore the functional and molecular characteristics of clusters.

Specifically, in step 3 of the above procedure, users have the option to input the cell cluster labels in the target data (the default option in ClusterDE), or they can allow the target data to be re-clustered by ClusterDE. If the default option is used, then ClusterDE performs clustering on the synthetic null data only, and the target DE scores will be calculated based on the input cell clusters. Otherwise, ClusterDE performs clustering on the target data and the synthetic null data in parallel, but the downside of this approach is that the target cell clusters might not be identical to the input cell clusters of users’ interest.

### ClusterDE method details

#### Notations for the double-dipping problem in post-clustering DE analysis

The target data is denoted by $Y=\left[Y_{ij}\right]\in N_{\geq 0}^{n\times m}$, a cell-by-gene UMI count matrix with n cells as rows, m genes as columns, and $Y_{ij}$ as the UMI count of gene j=1,…,m in cell i=1,…,n. We treat each cell i as an observation, which is an m-dimensional vector $Y_{i}={\left(Y_{i1},\cdots ,Y_{im}\right)}^{⊤}$.

In our formulation of the post-clustering DE problem, the n cells belong to two latent cell types and are partitioned into two clusters by a clustering algorithm. Accordingly, we use $Z_{i}\in \{0,\;1\}$ to denote cell i’s latent cell type.

We define the “ideal DE test” as the one that decides whether a gene has equal mean expression in two cell types. For gene j, we assume that ${\left\{\left(Y_{ij}|Z_{i}=0\right)\right\}}_{i=1}^{n}$ share the same mean denoted by $\mu_{0j}=E\left[Y_{ij}|Z_{i}=0\right]$, and ${\left\{\left(Y_{ij}|Z_{i}=1\right)\right\}}_{i=1}^{n}$ share the same mean denoted by $\mu_{1j}=E\left[Y_{ij}|Z_{i}=1\right]$. Then the ideal DE test has the following null hypothesis $H_{0j}$ and alternative hypothesis $H_{1j}$:

$$H_{0j}\;:\mu_{0j}=\mu_{1j}\;\;\text{vs.}\;\;H_{1j}\;:\mu_{0j}\neq \mu_{1j}.$$

Hence, gene j is a true DE gene if and only if $H_{0j}$ does not hold. When all n cells belong to one cell type only, all m null hypotheses, $H_{01},\ldots ,H_{0m}$, hold simultaneously.

However, since $Z_{i}$ ‘s are unobserved, standard single-cell data analysis partitions cells into two clusters using a clustering algorithm g (e.g., the Louvain algorithm in Seurat) applied to Y. We use ${\hat{Z}}_{i}=g_{Y}\left(Y_{i}\right)\in \{0,\;1\}$ to denote cell i’s cluster membership, where $g_{Y}\;:\left\{Y_{1},\ldots ,Y_{n}\right\}\to \{0,\;1\}$ is the clustering function, constructed from the clustering algorithm g and the data Y, that maps a cell’s gene expression vector to a cluster membership.

After cell clustering, standard single-cell analysis performs a DE test for each gene based on $Y_{1},\ldots ,Y_{n}$ and ${\hat{Z}}_{1},\ldots ,{\hat{Z}}_{n}$. In other words, the data Y is used twice (in clustering and DE analysis), referred to as the “double-dipping (DD) issue.” The standard post-clustering DE analysis used in the Seurat pipeline has the DD issue, and it tackles a statistical test different from the ideal DE test. Specifically, for gene j, we denote $\mu_{0j}^{DD}=E[Y_{ij}|{\hat{Z}}_{i}=0]$ and $\mu_{1j}^{DD}=E[Y_{ij}|{\hat{Z}}_{i}=1]$, two parameters that are the same for all i=1,…,n. Then, the post-clustering DE method in Seurat corresponds to the following null hypothesis $H_{0j}^{DD}$ and alternative hypothesis $H_{1j}^{DD}$:

$$H_{0j}^{DD}\;:\mu_{0j}^{DD}=\mu_{1j}^{DD}\;\;\text{vs.}\;\;H_{1j}^{DD}\;:\mu_{0j}^{DD}\neq \mu_{1j}^{DD}.$$

Hence, gene j would be detected as a false-positive cell-type marker gene if $H_{0j}^{DD}$ is rejected but $H_{0j}$ holds, leading to an inflated FDR in identifying cell-type marker genes. Fig. S16 provides a toy example illustration of this issue.

#### ClusterDE step 1: synthetic null generation

Previous findings indicate that, in a single cell type, each gene’s UMI counts can be fitted well by a negative binomial (NB) distribution [30–32], and all genes’ UMI counts can be well approximated by a multivariate NB (MVNB) distribution specified by the Gaussian copula [18]. Based on these findings, in ClusterDE, the null model that indicates a single “hypothetical” cell type is an MVNB distribution specified by the Gaussian copula. In ClusterDE step 1, the null model would be fitted on the real data Y by scDesign3 [18], and subsequently, synthetic null data would be sampled from the fitted null model. The intuition behind this null model is that cells of a single cell type constitute a sample from a homogenous population, in which every gene’s marginal count distribution is NB, and the gene-gene correlation structure is specified by the Gaussian copula. In addition, since scDesign3 supports many other choices of marginal distributions, ClusterDE can also generate synthetic null data from multivariate Gaussian, multivariate Poisson, multivariate Zero-Inflated Poisson, and multivariate Zero-Inflated Negative Binomial distribution.

Note that the idea of fitting a null model on real data, regardless of whether the real data was generated from the null model, is the core idea of the commonly used likelihood-ratio test in statistics [33], in which the maximum likelihood under the null hypothesis is estimated from the real data. Then the null maximum likelihood is compared with the alternative maximum likelihood, which is also estimated from the real data under a more flexible alternative hypothesis. Finally, the null hypothesis is only rejected if the null maximum likelihood is significantly smaller than the alternative maximum likelihood. ClusterDE generalizes this idea by sampling synthetic null data from the null model fitted by maximum likelihood estimation on the real data, so any clustering-followed-by-DE pipeline, however complicated, can be applied to the synthetic null data in parallel to the real data. Then a contrastive strategy can identify trustworthy DE genes as those whose DE scores are significantly higher from the real data than the synthetic null data.

Fig. S1 illustrates the synthetic null generation process detailed below. In the R package ClusterDE, this step 1 is implemented by the R package scDesign3 (version 0.99.0) [18].

##### 1. The null model: MVNB specified by the Gaussian copula

Under the null model, we assume that $Y_{ij}$, gene j ‘s UMI count in cell i, independently follows the $NB\left(\mu_{j},\;\sigma_{j}\right)$ distribution with the probability mass function:

$$P\left(Y=y;\;\mu_{j},\sigma_{j}\right)=\frac{\Gamma \left(y\;+\;\frac{1}{\sigma_{j}}\right)}{\Gamma \left(\frac{1}{\sigma_{j}}\right)\Gamma (y\;+\;1)}{\left(\frac{1}{1\;+\;\sigma_{j}\mu_{j}}\right)}^{\frac{1}{\sigma_{j}}}{\left(\frac{\sigma_{j}\mu_{j}}{1\;+\;\sigma_{j}\mu_{j}}\right)}^{y};\;y\in \{0,\;1,\;2,\cdots \},$$

where $\mu_{j}$ and $\sigma_{j}$ are the mean and dispersion parameters of the NB distribution. That is,

$$Y_{1j},\cdots ,Y_{nj}\overset{\;\text{i.i.d.}\;}{\sim }NB\left(\mu_{j},\sigma_{j}\right),$$

with “i.i.d.” short for “independent and identically distributed,” meaning that the n cells’ counts for gene j represent a random sample from $NB\left(\mu_{j},\;\sigma_{j}\right)$.

Denoting $F_{j}$ as the cumulative distribution function (CDF) of $NB\left(\mu_{j},\sigma_{j}\right)$, j=1,…,m, the MVNB distribution specified by the Gaussian copula is

$${\left(\Phi^{-1}\left(F_{1}\left(Y_{11}\right)\right),\cdots ,\Phi^{-1}\left(F_{m}\left(Y_{1m}\right)\right)\right)}^{⊤},\cdots ,{\left(\Phi^{-1}\left(F_{1}\left(Y_{n1}\right)\right),\cdots ,\Phi^{-1}\left(F_{m}\left(Y_{nm}\right)\right)\right)}^{⊤}\overset{\;\text{i.i.d.}\;}{\sim }N_{m}(0,\;R),$$

where Φ is the CDF of the standard Gaussian distribution N(0,1), and $N_{m}(0,\;R)$ is an m-dimensional Gaussian distribution with an m-dimensional 0 mean vector and an m-by-m correlation matrix R (which is also the covariance matrix because all m Gaussian variables have unit variances). This null model assumes that, after each gene is transformed to a standard Gaussian random variable, the n cells represent a random sample from an m-dimensional Gaussian distribution with zero means, unit variances, and a correlation matrix R.

In summary, the null model parameters include ${\left\{\mu_{j},\;\sigma_{j}\right\}}_{j=1}^{m}$ and R.

##### 2. Fitting the null model to real data (parameter estimation)

First, the parameters ${\left\{\mu_{j},\;\sigma_{j}\right\}}_{j=1}^{m}$ are estimated by employing the maximum likelihood estimation for the m NB distributions: using $Y_{1j},\ldots ,Y_{nj}$ to estimate $\mu_{j}$ and $\sigma_{j}$ as ${\hat{\mu }}_{j}$ and ${\hat{\sigma }}_{j}$, respectively, j=1,…,m. Based on ${\left\{{\hat{\mu }}_{j},\;{\hat{\sigma }}_{j}\right\}}_{j=1}^{m}$, the corresponding CDFs are denoted as ${\hat{F}}_{1},\ldots ,{\hat{F}}_{m}$.

Second, to estimate R, each $Y_{ij}$ is first transformed as $U_{ij}=V_{ij}\cdot {\hat{F}}_{j}\left(Y_{ij}\right)\;+\;\left(1-V_{ij}\right)\cdot {\hat{F}}_{j}\left(Y_{ij}\;+\;1\right)$, where $V_{ij}\overset{\text{i.i.d.}}{\sim }Uniform[0,\;1]$, so that $U_{ij}\sim Uniform[0,\;1]$. This procedure is referred to as the “distribution transform” to convert a discrete random variable $Y_{ij}$ to a continuous Uniform[0,1] random variable [34]. Then, R is estimated as the sample correlation matrix of

$${\left(\Phi^{-1}\left(U_{11}\right),\cdots ,\Phi^{-1}\left(U_{1m}\right)\right)}^{⊤},\cdots ,{\left(\Phi^{-1}\left(U_{n1}\right),\cdots ,\Phi^{-1}\left(U_{nm}\right)\right)}^{⊤}$$

and denoted as $\hat{R}$.

In summary, the fitted null model parameters include ${\left\{{\hat{\mu }}_{j},{\hat{\sigma }}_{j}\right\}}_{j=1}^{m}$ and $\hat{R}$.

##### 3. Sampling from the fitted null model (synthetic null data generation)

First, n Gaussian vectors of m dimensions are independently sampled $N_{m}(0,\;\hat{R})$ as

$${\left({\tilde{Z}}_{11},\cdots ,{\tilde{Z}}_{1m}\right)}^{⊤},\cdots ,{\left({\tilde{Z}}_{n1},\cdots ,{\tilde{Z}}_{nm}\right)}^{⊤}.$$

Second, The n Gaussian vectors are converted to NB count vectors as

$${\tilde{Y}}_{1}\;:={\left({\hat{F}}_{1}^{-1}\left(\Phi \left({\tilde{Z}}_{11}\right)\right),\cdots ,{\hat{F}}_{m}^{-1}\left(\Phi \left({\tilde{Z}}_{1m}\right)\right)\right)}^{⊤},\cdots ,{\tilde{Y}}_{n}\;:={\left({\hat{F}}_{1}^{-1}\left(\Phi \left({\tilde{Z}}_{n1}\right)\right),\cdots ,{\hat{F}}_{m}^{-1}\left(\Phi \left({\tilde{Z}}_{nm}\right)\right)\right)}^{⊤},$$

which represent the n synthetic null cells, each of which contains m genes’ synthetic null counts sampled from the null model.

In summary, the real data is an n-by-m count matrix Y with the n real cells $Y_{1},\ldots ,Y_{n}$ as the rows, while the synthetic null data is also an n-by-m count matrix $\tilde{Y}$ with the n synthetic null cells ${\tilde{Y}}_{1},\ldots ,{\tilde{Y}}_{n}$ as the rows. Note that there is no one-to-one correspondence between the real cells and the synthetic null cells because the synthetic null cells are independently sampled from the null model.

#### ClusterDE step 2: cell clustering

While ClusterDE allows any clustering algorithm, to align with the most common practice, we used the R package Seurat (version 4.2.0) for cell clustering in the results. That is, we applied the default Seruat clustering to the target data and the synthetic null data in parallel, obtaining two cell clusters in each dataset respectively.

Specifically, the default Seurat clustering includes the following steps applied to both the target data and the synthetic null data, each of which is stored as a Seurat object. We denote each Seurat object as Seurat.obj.

1. Normalize each cell to have a total count of 10,000; then perform log(normalized count + 1) transformation.

NormalizeData(Seurat.obj, normalization.method = “LogNormalize”, scale.factor = 10000)
Select 2,000 highly variable genes.

FindVariableFeatures (Seurat.obj, selection.method = “vst”, nfeatures = 2000)
Scale the data.

ScaleData (Seurat.obj)
Run PCA on the data.

RunPCA (Seurat.obj, features = VariableFeatures () )
Compute cells’ k-nearest neighbors.

FindNeighbors (Seurat.obj, dims = 1:30, nn.method = “rann”, k param = 20)
Perform Louvain clustering on the cells.

FindClusters (Seurat.obj, resolution)
  Since the Louvain clustering cannot pre-specify the cluster number, we tried resolutions starting from the default resolution of 0.5 and adjusted the resolution until two clusters were found.

After applying the above clustering procedure, we obtained the cluster labels ${\hat{Z}}_{1},\ldots ,{\hat{Z}}_{n}$ from the target data Y, and ${\tilde{Z}}_{1},\ldots ,{\tilde{Z}}_{n}$ from the synthetic null data $\tilde{Y}$, respectively, where ${\hat{Z}}_{i},{\tilde{Z}}_{i}\in \{0,\;1\}$, i=1,…,n. Again, there exists no one-to-one correspondence between ${\hat{Z}}_{1},\ldots ,{\hat{Z}}_{n}$ and ${\tilde{Z}}_{1},\ldots ,{\tilde{Z}}_{n}$.

#### ClusterDE step 3: DE analysis

ClusterDE allows any DE test. In the results, we used five DE tests included in the Seurat function FindMarkers, including the Wilcoxon rank-sum test (Wilcoxon, the default test), t-test, negative binomial generalized linear model (NB-GLM), logistic regression model predicting cluster membership with likelihood-ratio test (LR), and likelihood-ratio test for single cell gene expression (bimod, ref [35]).

Given a DE test, on the target data, ClusterDE computes a P value $P_{j}$ for each gene j for testing the null hypothesis $H_{0j}^{DD}\;:\mu_{0j}^{DD}=\mu_{1j}^{DD}$, where $\mu_{0j}^{DD}=E[Y_{ij}|{\hat{Z}}_{i}=0]$ and $\mu_{1j}^{DD}=E[Y_{ij}|{\hat{Z}}_{i}=1]$. Then the target DE score of gene j is defined as $S_{j}\;:=-{log}_{10}P_{j}$.

In parallel, on the synthetic null data, ClusterDE calculates a *P* value ${\tilde{P}}_{j}$ for each gene j for testing the null hypothesis ${\tilde{H}}_{0j}^{DD}\;:{\tilde{\mu }}_{0j}^{DD}={\tilde{\mu }}_{1j}^{DD}$, where ${\tilde{\mu }}_{0j}^{DD}=E\left[{\tilde{Y}}_{ij}|{\tilde{Z}}_{i}=0\right]$ and ${\tilde{\mu }}_{1j}^{DD}=E\left[{\tilde{Y}}_{ij}|{\tilde{Z}}_{i}=\right.1]$. Then the null DE score of gene j is defined as ${\tilde{S}}_{j}\;:=-{log}_{10}{\tilde{P}}_{j}$.

In summary, the m genes have the target DE scores $S_{1},\ldots ,S_{m}$ and the null DE scores ${\tilde{S}}_{1},\ldots ,{\tilde{S}}_{m}$.

#### ClusterDE step 4: FDR control

Given the target DE scores $S_{1},\ldots ,S_{m}$ and the null DE scores ${\tilde{S}}_{1},\ldots ,{\tilde{S}}_{m}$, we use the FDR-control method Clipper to identify DE genes given a target FDR threshold q∈(0,1) [19]. Given a set of identified DE genes, the FDR is defined as

$$FDR\;:=E\left[\frac{\#\;\text{false}\;\text{discoveries}\;}{\#\;\text{discoveries}\;\vee \;1}\right],$$

where a∨b is defined as the maximum of two numbers a and b.

To ensure a valid FDR control, Clipper requires each gene to have a contrast score such that the true non-DE genes have contrast scores symmetric about zero. In ClusterDE, gene j’s contrast score $C_{j}$ is defined as

$$C_{j}\;:=S_{j}-{\tilde{S}}_{j}.$$

Then ClusterDE uses Clipper to find a contrast score cutoff T within 𝒞 (i.e., the set of non-zero contrast score values) given the target FDR threshold q:

$$T\;:=min\left\{t\in 𝒞\;:\;\frac{\left|\left\{j\;:\;C_{j}\;\leq \;-t\right\}\right|\;+\;1}{\left|\left\{j\;:\;C_{j}\;\geq \;t\right\}\right|\;\vee \;1}\leq q\right\}$$

and outputs $\left\{j\in \left\{1,\cdots ,m\right\}\;:C_{j}\geq T\right\}$ as discoveries. Here |A| defines the size of a set A. The FDR control of this contrast-score thresholding procedure was from the knockoffs method [21].

Under the assumption that the majority of genes are non-DE genes, we would expect that the distribution of all genes’ contrast scores has a mode at zero, so the symmetry requirement of Clipper is satisfied. That is, in the ideal scenario, slightly less than 50% of all genes’ contrast scores should be negative. However, in some real data scenarios, this symmetry requirement might not hold. For example, the contrast score distribution might have a positive mode such that too few contrast scores are negative, leading to inflated false discoveries made by Clipper. Or it could be that the contrast score distribution has a negative mode such that too many contrast scores are negative, leading to a loss of statistical power. Hence, in practice, ClusterDE verifies the symmetry assumption by employing Yuen’s trimmed mean test (using the function yuen.t.test() from the R package PariedData (version 1.1.1)). This test examines the symmetry of the contrast score distribution after excluding the smallest 10% and largest 10% of the contrast scores.

If symmetry is rejected by Yuen’s trimmed mean test, ClusterDE applies an adjustment to the contrast score distribution so that the symmetry requirement can approximately hold. In detail, ClusterDE applies the “robust fitting of linear models” (using the function rlm() from the R package MASS (version 7.3–60)) to adjust the null DE scores; that is, a linear model is fitted between the target DE scores (the response variable y) and the null DE scores (the explanatory variable x), and the fitted values (the predicted response variable $\overset{ˆ}{y}$) are taken as the adjusted null DE scores. Then the adjusted contrast scores, defined as the differences between the target DE scores and the adjusted null DE scores, would better satisfy the symmetry requirement.

Since we would like to be conservative regarding the adjustment of contrast scores, ClusterDE uses the one-sided (“greater than”) Yuen’s trimmed mean test at the significance level of 0.001. Hence, adjustment is performed only when too few contrast scores are negative, a scenario that would lead to inflated false discoveries made by Clipper.

### Implementation of the TN test and Countsplit

We compared ClusterDE with two existing methods—the TN test (ref [5]) and Countsplit (ref [6])—that attempted to address the double-dipping issue in post-clustering DE analysis.

For the TN test, we used the Python module truncated-normal (version 0.4). We followed the GitHub tutorial for the implementation (https://github.com/jessemzhang/tn_test/blob/master/experiments/experiments_pbmc3k.ipynb). In the clustering step, we used the same procedure as in ClusterDE step 2. In the DE analysis step, unlike ClusterDE and Countsplit, the TN test has its own DE test, so we did not use any DE tests included in the R package Seurat (version 4.2.0).

For Countsplit [6], we used the R package countsplit (version 1.0) to split the original count matrix into a training matrix (for clustering) and a test matrix (for DE analysis). In the clustering step, we used the same procedure as in ClusterDE step 2. In the DE analysis step, we used the five DE tests included in the R package Seurat (version 4.2.0).

### Alternative strategies for synthetic null generation

Although the model-X knockoffs method was developed for selecting features in a multivariate predictive model (e.g., the Lasso) [22], not for marginal DE tests (where each feature is examined separately), we compared model-X knockoffs to ClusterDE because both methods use the real-data-based negative control idea.

For a direct implementation of the model-X knockoffs method on the post-clustering DE analysis, we used the R package knockoff (version 0.3.6) to construct the knockoff data (i.e., the negative control) and used the default glmnet method for binary logistic regression (where the cluster labels are considered as the response variable y, and the genes are considered as the features) to select features as DE genes. We test this approach on 50 simulated datasets with log⁡FC=2.6 (see “Simulation design with two cell types and 200 DE genes) and found that it always selected 0 DE genes (i.e., the power was always 0).

Moreover, we used the knockoff data constructed above and the permuted data (where each gene was independently permuted across all cells) as two alternative synthetic null generation strategies (alternatives to scDesign3) in ClusterDE step 1. Our results on the simulated datasets indicate that scDesign3 led to more solid FDR control and better statistical power than these two alternative strategies for the synthetic null generation (Fig. S8).

### Simulation designs

To benchmark post-clustering DE methods in terms of the FDR and statistical power, we needed ground truths of DE genes and non-DE genes. Hence, we used the R package scDesign3 (version 0.99.0) [18] to generate realistic synthetic scRNA-seq data containing true DE genes and non-DE genes, based on the model parameters learned from real scRNA-seq data. Under each simulation setting, we generated 200 synthetic replicates.

For each replicate, we simulated a dataset with n=998 cells and m=9239 genes, the same dimensions as those of the naïve cytotoxic T cells in the Zhengmix4eq dataset [20] after the default Seurat preprocessing step that removed the genes expressed at very low levels. In the following, we let i and j denote the indices of cells and genes, respectively, i=1,…,n;j=1,…,m.

The first step was to estimate the following model parameters from the naïve cytotoxic T cells in the Zhengmix4eq dataset by scDesign3 [18]. For details of the model formulation, please refer to the previous section “ClusterDE step 1: synthetic null generation.”

- Per-gene NB mean parameter $\mu_{j}\in R^{\;+\;},\;j=1,\cdots ,m$;
- Per-gene NB dispersion parameter $\sigma_{j}\in R^{\;+\;},\;j=1,\cdots ,m$;
- Gene-gene Gaussian copula correlation matrix $R\in [0,\;1{]}^{m\times m}$.

Given the mode model parameters include ${\left\{{\hat{\mu }}_{j},{\hat{\sigma }}_{j}\right\}}_{j=1}^{m}$ and $\hat{R}$, the next steps belonged to two settings: (1) one cell type with zero true DE genes; (2) two cell types with 200 true DE genes.

#### Simulation setting with one cell type and zero true DE genes

All of the n=998 cells were simulated from one cell type with an MVNB distribution specified by the Gaussian copula, whose correlation matrix was $\hat{R}$, so gene j ‘s counts followed the NB distribution with mean ${\hat{\mu }}_{j}$ and dispersion ${\hat{\sigma }}_{j}$, j=1,⋯,m. For the simulation details, please refer to the previous section “ClusterDE step 1: synthetic null generation.” The R package scDesign3 (ref [18]) was used to simulate a cell-by-gene count matrix $Y\in N_{\geq 0}^{n\times m}$, which was used as the one-cell-type target data (Fig. 1p) in simulation studies.

#### Simulation setting with two cell types and 200 true DE genes

All of the n=998 cells were designed to belong to two cell types, with each cell type having its own MVNB distribution specified by the Gaussian copula. For each replicate, we randomly specified 200 true DE genes to have different mean parameters $\mu_{j}^{0}$ and $\mu_{j}^{1}$ based on the estimate ${\hat{\mu }}_{j}$.

For the two cell types, we simulated three cell-type size ratios r∈{1,4,9} such that cells $i=1,\ldots ,\left[\frac{n}{r+1}\right]$ were designed to be of cell type 0, and cells $i=\left[\frac{n}{r+1}\right]+1,\ldots ,n$ were designed to be of cell type 1. For each replicate, the set 200 true DE genes were specified with the index set $J_{DE}\subset \{1,\cdots ,m\}$.

For each specified true DE gene $j\in J_{DE}$, we set its mean parameter in cell type 0 as the estimate, i.e., $\mu_{j}^{0}={\hat{\mu }}_{j}$. Then we modified its mean parameter in cell type1, $\mu_{j}^{1}$, using a pre-specified log fold change logFC with a 50% probability of up-regulation and a 50% probability of down-regulation:

$$\mu_{j}^{1}=\left\{\begin{array}{ll} {\hat{\mu }}_{j}\times 2^{logFC}, & \;\text{if}\;Z_{j}=1\\ {\hat{\mu }}_{j}\times 2^{-logFC}, & \;\text{if}\;Z_{j}=0 \end{array},\;\;\text{with}\;Z_{j}\sim Ber(0.5),\;j\in J_{DE}.\right.$$

For the remaining true non-DE genes, we set

$$\mu_{j}^{0}=\mu_{j}^{1}={\hat{\mu }}_{j},\;j\in \{1,\cdots ,m\}\backslash J_{DE}.$$

The parameter log⁡FC determines the differences between the two cell types, and it is expected to have an inverse relationship with the severity level of double dipping (that is, the more different the two cell types, the less severe the double dipping). Hence, we simulated two cell types with a sequence of logFC values

log⁡FC=1.05,1.1,⋯,1.95,2,2.1,⋯,2.9,3.

For each log⁡FC value, we simulated cells from cells 0 and 1, each with an MVNB distribution specified by the Gaussian copula, whose correlation matrix was $\hat{R}$. That is, gene j’s counts in cell types 0 and 1 followed NB distributions with different mean parameters $\mu_{j}^{0}$ and $\mu_{j}^{1}$, respectively, and the same dispersion parameter ${\hat{\sigma }}_{j}$, j=1,⋯,m. For the simulation details, please refer to the previous section “ClusterDE step 1: synthetic null generation.” The R package scDesign3 (ref [18]) was used to simulate a cell-by-gene count matrix $Y\in N_{\geq 0}^{n\times m}$, which was used as the two-cell-type target data (Fig. S2) in simulation studies.

### Real data analysis

#### Collection of real data

We collected five scRNA-seq datasets of cell lines, including the three datasets of A549, H2228, and HCC827 from the study [24] and downloaded from the link https://github.com/LuyiTian/sc_mixology/tree/master/data, and the two datasets HEK293T and JUKART from the study [23] and downloaded from https://cf.10xgenomics.com/samples/cell-exp/1.1.0/jurkat/jurkat_filtered_gene_bc_matrices.tar.gz and https://cf.10xgenomics.com/samples/cell-exp/1.1.0/293t/293t_filtered_gene_bc_matrices.tar.gz.

We also collected eight peripheral blood mononuclear cell (PBMC) datasets from the study [25], which were downloaded from https://github.com/satijalab/seurat-data. The datasets were from the same biological sample with two technical replicates (Rep1/Rep2) measured by four protocols (10X Genomics Versions 2 and 3, Drop-seq, and inDrop). In each dataset, we selected the cells with cell type labels “CD14^+^monocytes” and “CD16^+^ monocytes.”

#### Data preprocessing

We filtered out lowly expressed genes. For the cell line datasets A549, H2228, and HCC827, we removed the genes expressed in fewer than 20% cells. For the cell line datasets HEK293T and JUKART, we removed genes expressed in fewer than 10% cells. For PBMC datasets, we removed genes expressed in fewer than 10% of the selected monocyte cells. When performing the default Seurat clustering, Seurat automatically removed the cells with fewer than three genes expressed and the genes expressed in fewer than 200 cells.

#### Dimensionality reduction and visualization

To visualize the high-dimensional single-cell data, we first applied the PF-logPF transformation to a cell-by-gene count matrix [36]. We then used the R package irlba (version 2.3.5.1) to calculate the top 50 principal components (PCs) of the transformed matrix. Next, we used the R package umap (version 0.2.10.0) to project the cells from the 50-dimensional PC space to the 2-dimensional UMAP space.

When comparing the target data and the synthetic null data, we calculated the PCs and UMAPs jointly by concatenating the two datasets so the target cells and synthetic null cells were projected to the same 2-dimensional UMAP space.

We used the R package ggplot2 (version 3.4.2) to make all plots.

For the UMAP visualizations in Fig. 2f, we truncated each gene’s normalized expression levels to be below the 99-th percentile to better visualize the gene expression pattern.

#### Gene set enrichment analysis

We used the R package clusterProfiler (4.4.4) to perform the gene set enrichment analysis (GSEA); the test method was fgsea, and the number of permutations was 100,000. The gene set “CD14^+^/CD16^+^ Monocyte Markers” was from the original study [25] and downloaded from https://bitbucket.org/jerry00/scumi-dev/raw/61f7f001d20b2fc8fa7c2f4f4147bff1b0d620d8/R/marker_gene/human_pbmc_marker.rda. The gene set “Housekeeping Genes”, HSIAO_HOUSEKEEPING_GENES, was downloaded from the Molecular Signature Database (MSigDB); the source study was [37].

#### Validity checks of the contrast scores of ClusterDE and the *P* values of Seurat, Countsplit, and the TN test

For ClusterDE, the major assumption is that the contrast scores of true non-DE genes are symmetric around zero. In Fig. S3 left, we checked the symmetry of the contrast scores of ClusterDE using the five DE tests (Wilcoxon, t-test, NB-GLM, LR, and bimod; corresponding to Fig. S3a–e left) in a simulated one-cell-type dataset where all genes are true non-DE genes (see Methods “Simulation setting with one cell type and zero true DE genes”; the dataset is one of the 200 synthetic replicates).

For Seurat, Countsplit, and the TN test methods, their FDR control validity requires that the *P* values of true non-DE genes follow the Uniform [0, 1] distribution. First, we divided the genes in the same simulated dataset into two groups by applying hierarchical clustering (using the default R function hclust()) to the estimated correlation matrix $\hat{R}$ used in the Gaussian copula. Due to the block pattern of $\hat{R}$ (Fig. 1 c), the two groups include the genes that are highly correlated and those that are not much correlated, respectively. We examined the *P* values of the genes in the two groups separately. Note that all genes in this simulated dataset are true non-DE genes. In Fig. S3 middle and right, we plotted the histograms of the *P* values and the quantile-quantile plots (Q-Q plots) of the negative log-transformed *P* values of Seurat, Countsplit (both using the five DE tests; corresponding to Fig. S3a–e) and the TN test (using its own test; the same panels plotted five time in Fig. S3a–e). We also used the R function KL.empirical (from the R package entropy (version 1.3.1)) to calculate the empirical Kullback–Leibler divergence (KL div.) between the *P*-value distribution and the theoretical Uniform [0, 1] distribution. A larger Kullback–Leibler divergence value represents a more severe violation of the *P*-value uniformity assumption. The results show that Countsplit and the TN test had close-to-uniform *P* values in the uncorrelated gene group, but their *P* values exhibited a severe departure from the uniform distribution in the correlated gene group.

## Supplementary Material

Supplement 1

## Data Availability

All datasets used in the study are publicly available. The pre-processed datasets are available at https://figshare.com/articles/dataset/ClusterDE_datasets/23596764.
