# Supplementary material for: ClusterDE: a post-clustering differential expression (DE) method robust to false-positive inflation caused by double dipping
Source: Res Sq. 2023 Aug 2:rs.3.rs-3211191. Preprint. [Version 1] doi: 10.21203/rs.3.rs-3211191/v1 (PMC10418557; doi:10.21203/rs.3.rs-3211191/v1)
Supplement: Supplement 1 [file NIHPPRS3211191V1-supplement-1.pdf]

Supplementary Figures

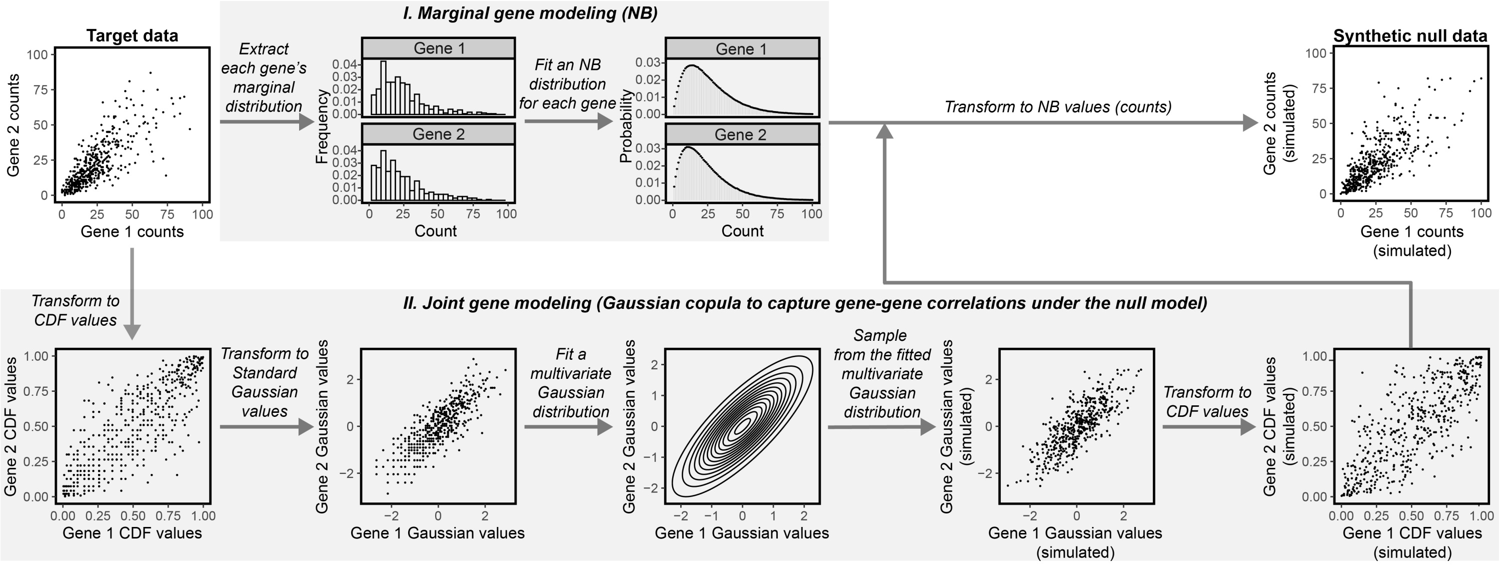

Figure S1: The generation process of synthetic null data from target data (top left) by scDesign3. We show the bivariate case (with two genes) for illustration purposes, and the real case is high-dimensional with thousands of genes. The null model consists of two parts: for marginal gene modeling, each gene's counts follow a negative binomial (NB) distribution; for joint gene modeling, the genes' dependence structure is specified by the Gaussian copula. For the marginal gene modeling part (top), a negative binomial (NB) distribution is fitted to each gene's counts in the target data, obtaining the two NB parameters (mean and dispersion) for each gene. For the joint gene modeling part (bottom), there are three steps. First, each gene's counts in the target data are transformed into cumulative distribution function (CDF) values, via the fitted NB distribution or the counts' empirical distribution (if the target cell number is large), so the gene's CDF values are uniform between 0 and 1. Second, each gene's CDF values are transformed into quantiles of the standard Gaussian $N(0,1)$ distribution. Third, a multivariate Gaussian distribution (a bivariate Gaussian distribution for illustration) is fitted to the transformed Gaussian values of the many genes whose correlations are to be modeled. The correlation matrix of the fitted multivariate Gaussian distribution specifies the Gaussian copula. After the marginal and joint modeling, the generation of the same genes' synthetic counts takes three steps. First, the genes' standard Gaussian values are jointly sampled from the fitted multivariate Gaussian distribution. Second, each gene's standard Gaussian values are transformed into the CDF values of the standard Gaussian distribution. Third, each gene's CDF values are transformed into quantiles of the gene's fitted NB distribution and thus become counts, which constitute the synthetic null data (top right).

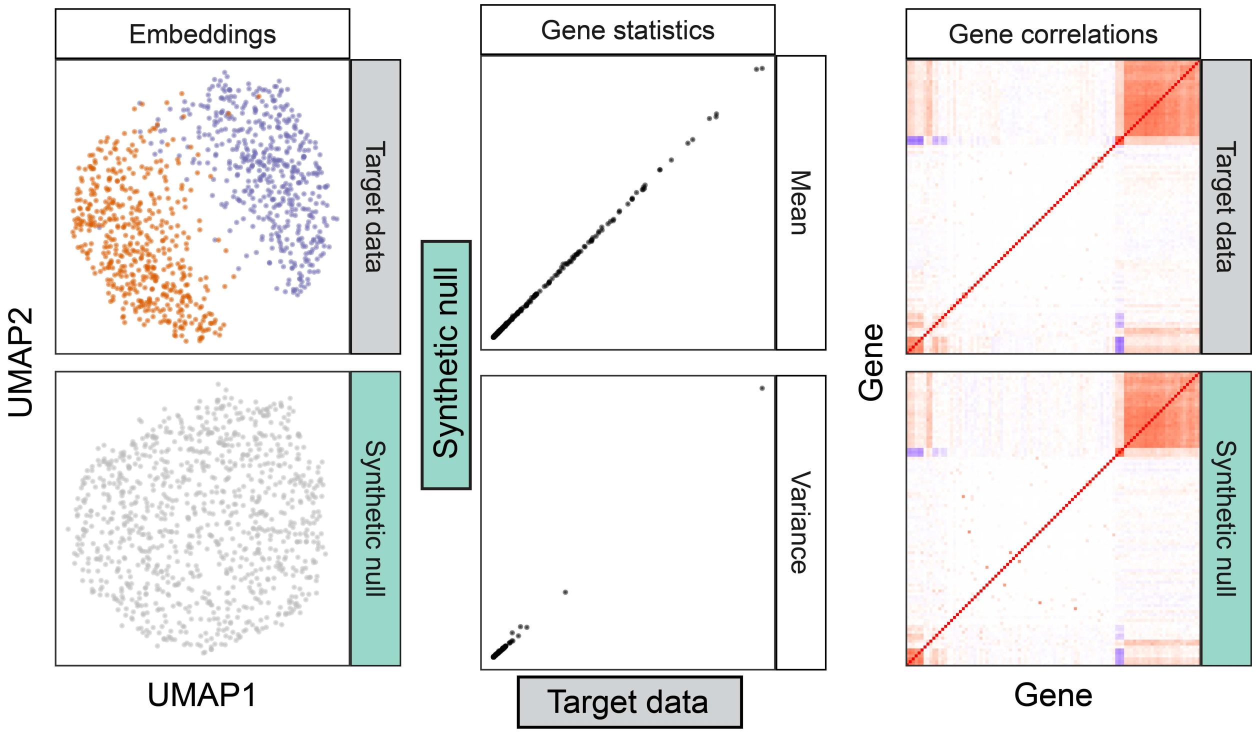

Figure S2: When the target data contains cells from two cell types (simulation; see Methods "Simulation design with one cell type and zero DE genes"), the synthetic null data generated by ClusterDE fills the gap between the two cell types but resembles the target data in other visual aspects of UMAP cell embeddings (left), per-gene expression mean and variance statistics (middle), and gene-gene correlations.

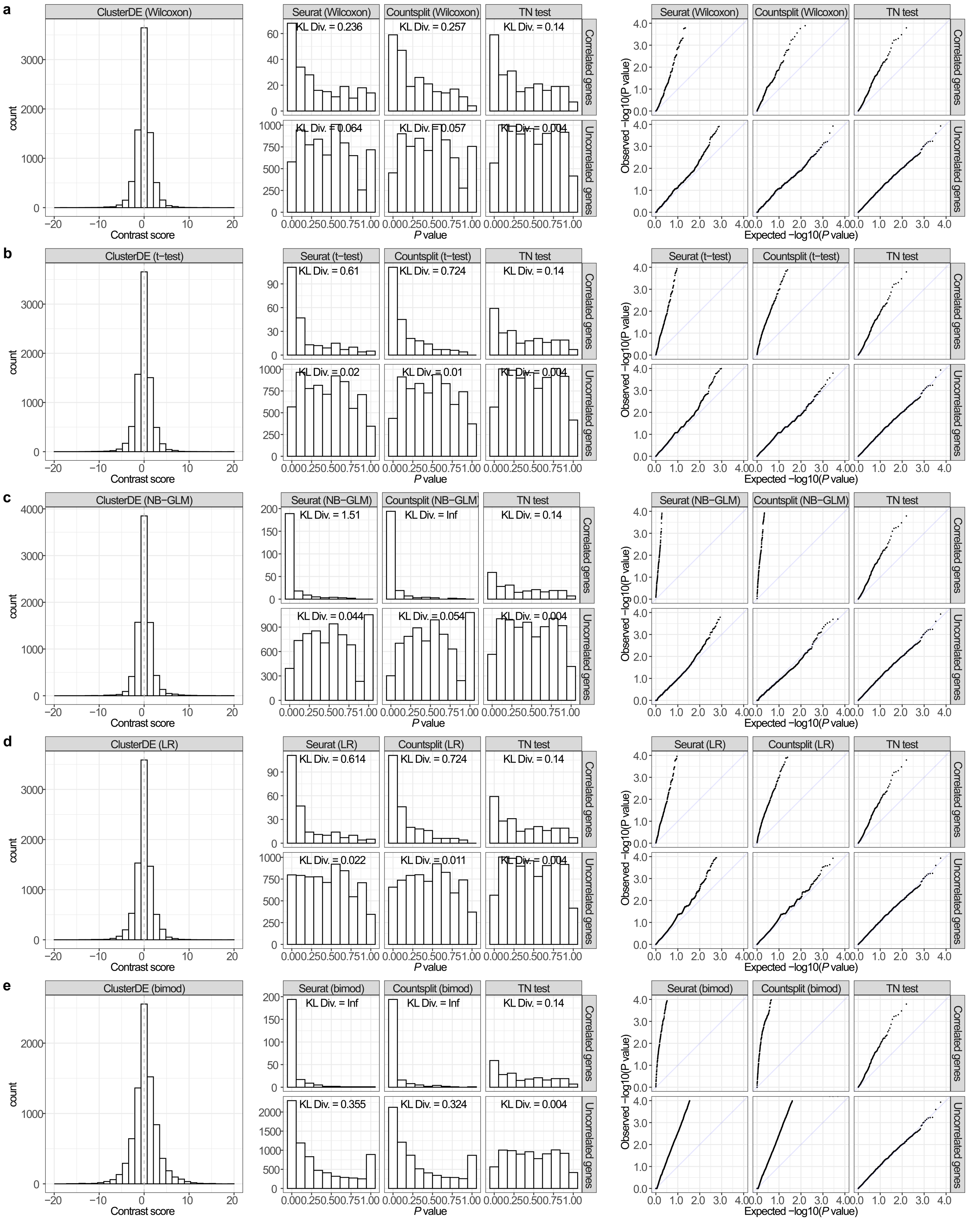

Figure S3: Validity checks of the contrast scores of ClusterDE and P values of Seurat, Countsplit, and the TN test on an exemplary one-cell-type dataset, which does not contain any true DE genes by the simulation design (see **Methods** "Simulation setting with one cell type and zero true DE genes"). The panels (rows) **a**–**e** represent the five DE tests in Seurat used in ClusterDE, Seurat, and Countsplit (see **Methods** "ClusterDE step 3: DE analysis"); since the TN test has its own DE test, its results are the same in the panels **a**–**e**. The first column shows that the ClusterDE contrast scores of all genes (true non-DE genes) are approximately symmetric around 0, which meets the assumption of ClusterDE for the FDR control. The second column shows the histograms of the P-values of the correlated genes (top) and the uncorrelated genes (bottom) from Seurat, Countsplit, and the TN test. A larger empirical Kullback-Leibler divergence (KL div.) between the P-value distribution and the theoretical $\mathrm{Uniform}[0,1]$ distribution represents a more severe violation of the P-value uniformity assumption. The results show that Countsplit and the TN test have close-to-uniform P values for the uncorrelated genes, but their P values exhibit a severe departure from the uniform distribution for the correlated genes. The third column contains the quantile-quantile plots of the negative log-transformed P values corresponding to the second column.

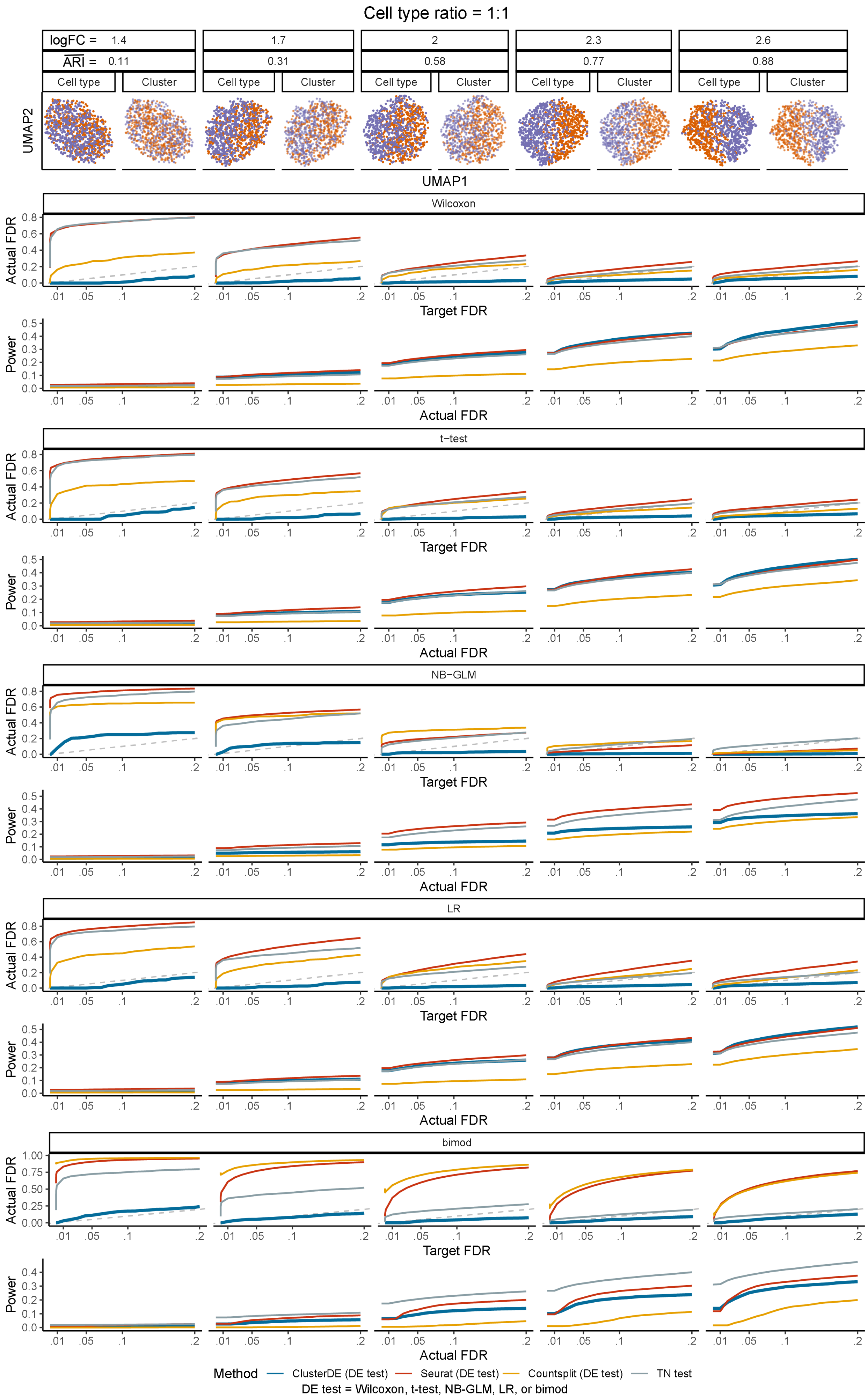

Cell type ratio = 1:1
logFC = 1.4
1.7
2
2.3
2.6
$\overline{\text{ARI}}$ = 0.11
0.31
0.58
0.77
0.88
Cell type
Cluster
UMAP2
UMAP1
Wilcoxon
t−test
NB−GLM
LR
bimod
Actual FDR
Target FDR
Power
Method
ClusterDE (DE test)
Seurat (DE test)
Countsplit (DE test)
TN test
DE test = Wilcoxon, t-test, NB-GLM, LR, or bimod

Figure S4: The FDRs and power of ClusterDE and the existing methods under various severity levels of double dipping when the two cell types have a size ratio of $1:1$. The log fold change (logFC) summarizes the average gene expression difference between the two cell types in simulation (see **Methods** "Simulation design with two cell types and 200 DE genes"). Corresponding to a small logFC, a small adjusted Rand index (ARI) represents a bad agreement between cell clusters and cell types, representing a more severe double-dipping issue. Across various severity levels of double dipping and the five DE tests, ClusterDE controls the FDRs under the target FDR thresholds (diagonal dashed line) and achieves comparable or higher power compared to the existing methods at the same actual FDRs.

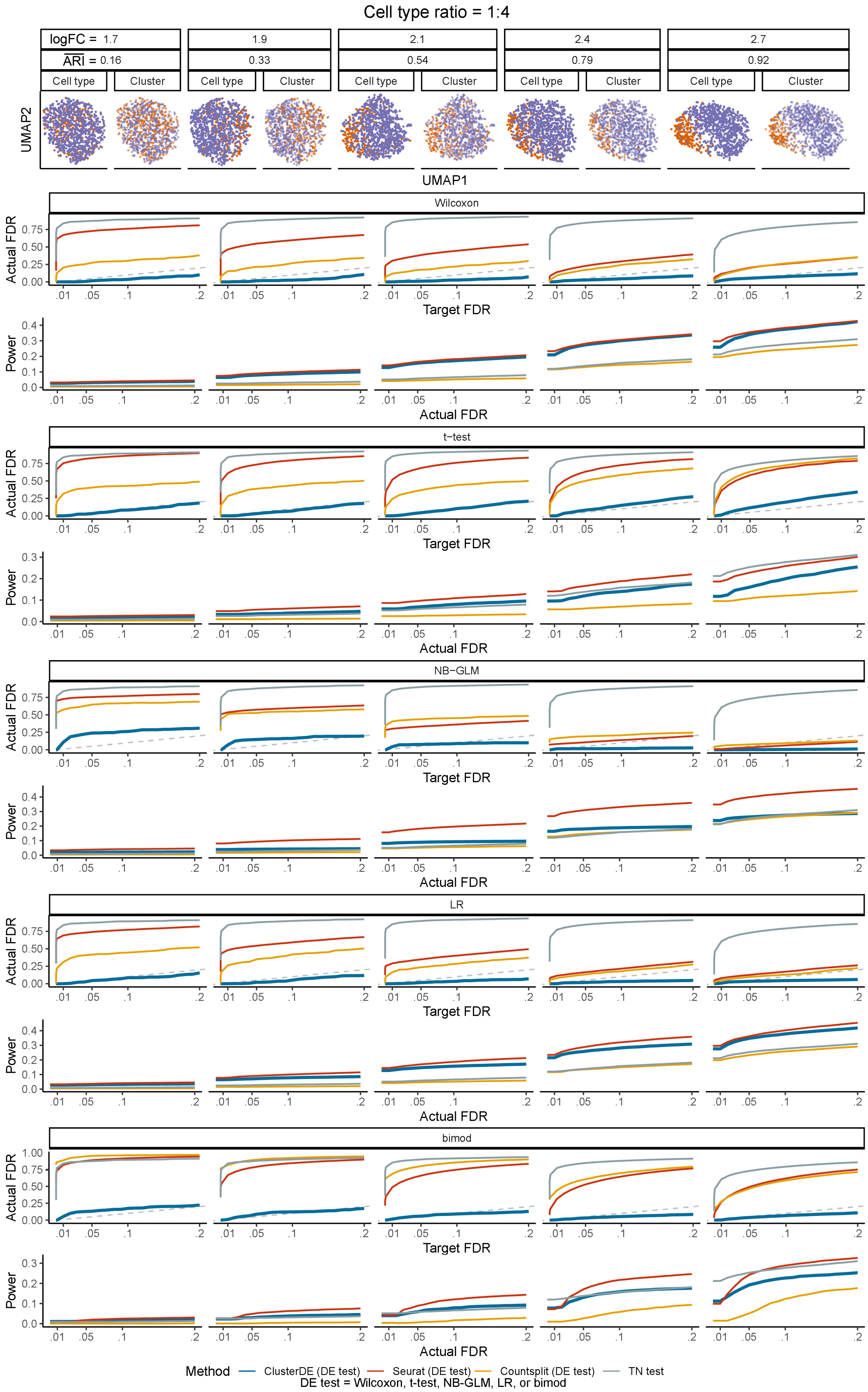
Cell type ratio = 1:4
logFC = 1.7
1.9
2.1
2.4
2.7
ARI = 0.16
0.33
0.54
0.79
0.92
Cell type
Cluster
UMAP2
UMAP1
Wilcoxon
t−test
NB−GLM
LR
bimod
Actual FDR
Target FDR
Power
0.00
0.25
0.50
0.75
1.00
0.0
0.1
0.2
0.3
0.4
.01
.05
.1
.2
Method
ClusterDE (DE test)
Seurat (DE test)
Countsplit (DE test)
TN test
DE test = Wilcoxon, t-test, NB-GLM, LR, or bimod

Figure S5: The FDRs and power of ClusterDE and the existing methods under various severity levels of double dipping when the two cell types have a size ratio of $1:4$. The log fold change (logFC) summarizes the average gene expression difference between the two cell types in simulation (see **Methods** "Simulation design with two cell types and 200 DE genes"). Corresponding to a small logFC, a small adjusted Rand index (ARI) represents a bad agreement between cell clusters and cell types, representing a more severe double-dipping issue. Across various severity levels of double dipping and the five DE tests, ClusterDE controls the FDRs under the target FDR thresholds (diagonal dashed line) and achieves comparable or higher power compared to the existing methods at the same actual FDRs.

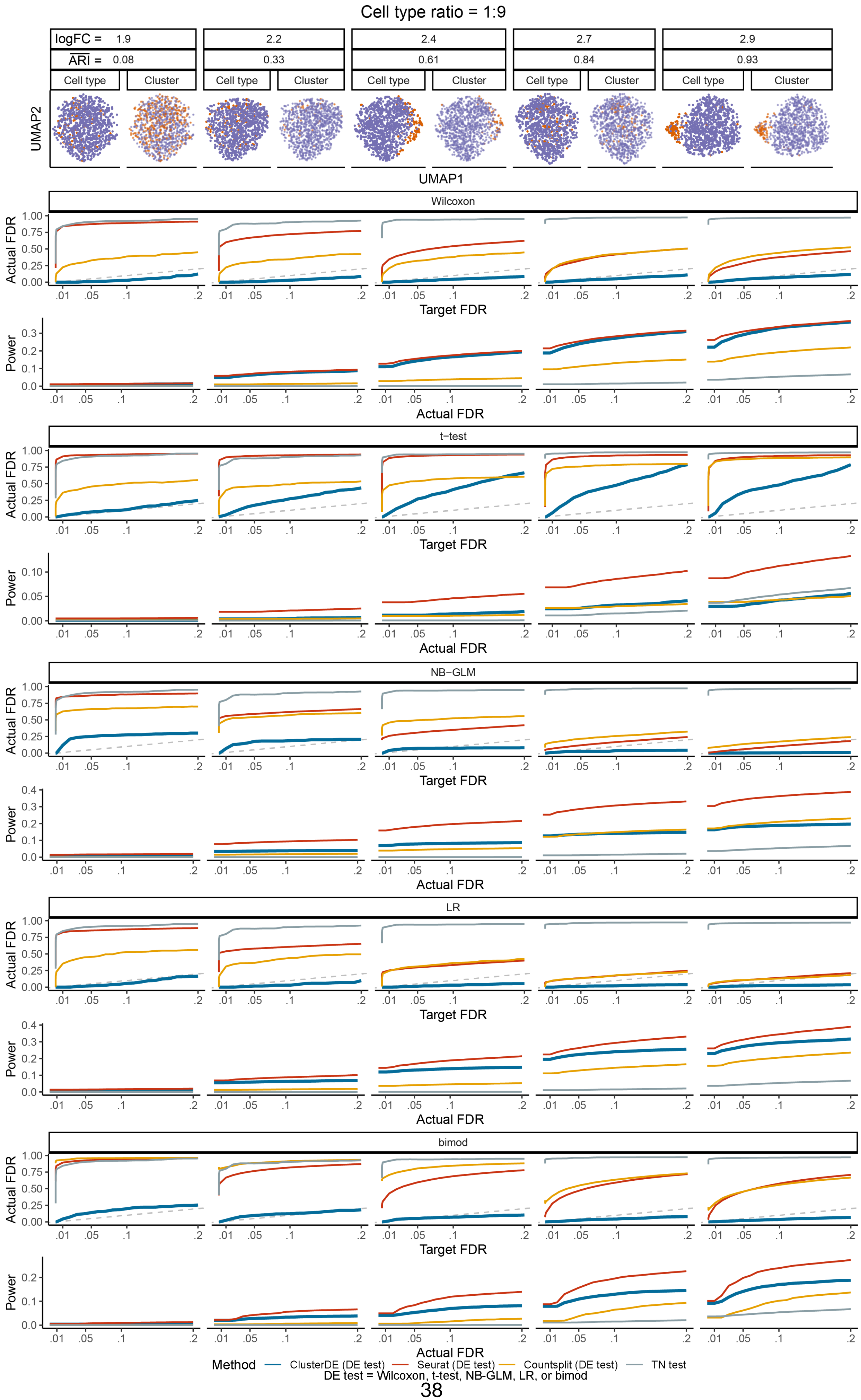

Cell type ratio = 1:9
logFC = 1.9
2.2
2.4
2.7
2.9
ARI = 0.08
0.33
0.61
0.84
0.93
Cell type
Cluster
UMAP2
UMAP1
Wilcoxon
t−test
NB−GLM
LR
bimod
Actual FDR
Target FDR
Power
Method
ClusterDE (DE test)
Seurat (DE test)
Countsplit (DE test)
TN test
DE test = Wilcoxon, t-test, NB-GLM, LR, or bimod

Figure S6: The FDRs and power of ClusterDE and the existing methods under various severity levels of double dipping when the two cell types have a size ratio of $1:9$. The log fold change (logFC) summarizes the average gene expression difference between the two cell types in simulation (see **Methods** “Simulation design with two cell types and 200 DE genes”). Corresponding to a small logFC, a small adjusted Rand index (ARI) represents a bad agreement between cell clusters and cell types, representing a more severe double-dipping issue. Across various severity levels of double dipping and the five DE tests, ClusterDE controls the FDRs under the target FDR thresholds (diagonal dashed line) and achieves comparable or higher power compared to the existing methods at the same actual FDRs.

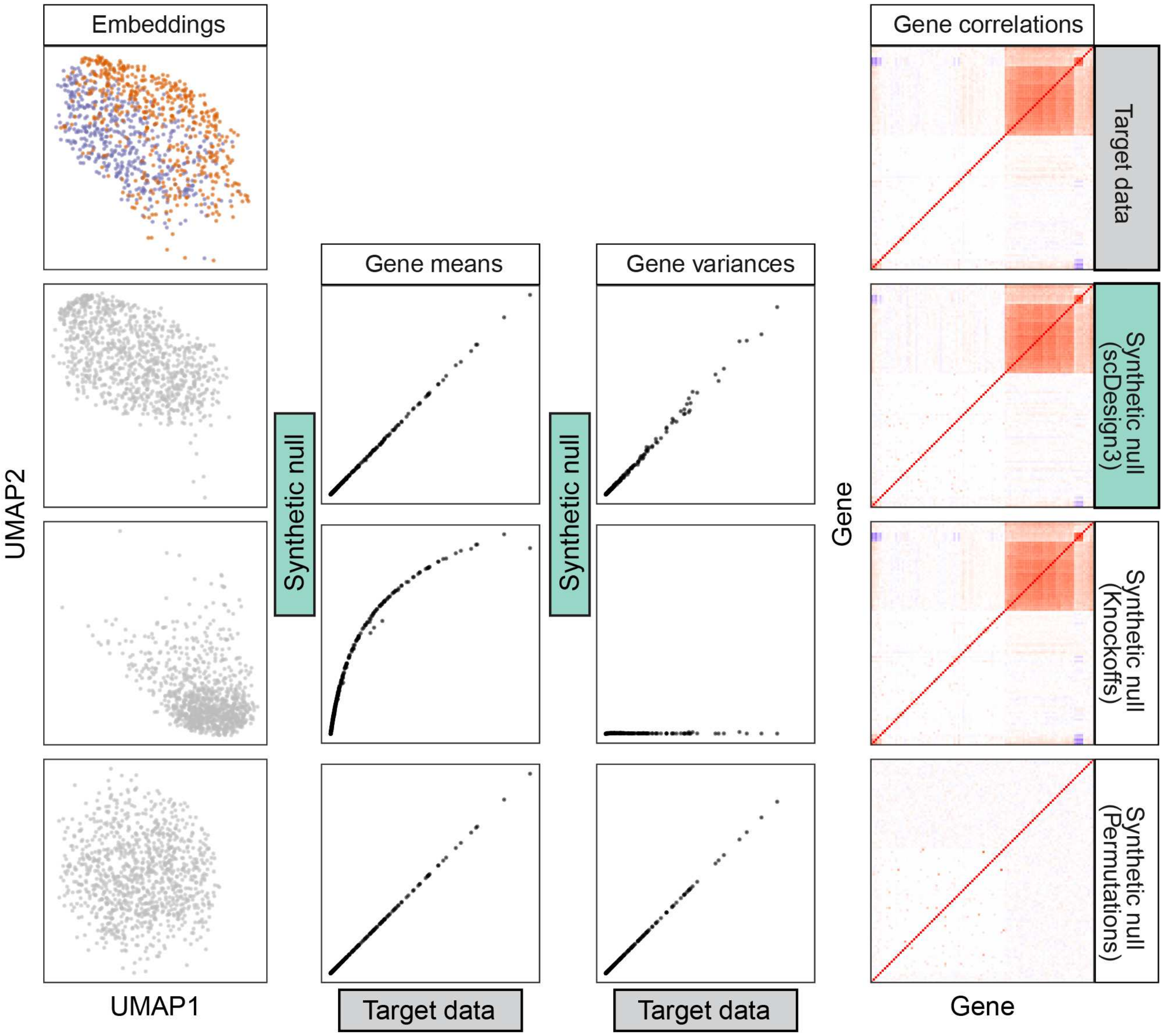

Figure S7: When the target data contains cells from two cell types (simulation; see Methods "Simulation design with one cell type and zero DE genes"), the synthetic null data generated by ClusterDE (second row) fills the gap between the two cell types but resembles the target data in other visual aspects of UMAP cell embeddings (left), per-gene expression mean and variance statistics (middle), and gene-gene correlations. In contrast, the synthetic null data generated by knockoffs (third row) and permutations (fourth row) do not resemble the target data. For the synthetic null data generated by knockoffs, it preserves the gene-gene correlations of the target data, but does not preserve per-gene expression mean and variance statistics. For the synthetic null data generated by permutations, it preserves per-gene expression mean and variance statistics of the target data, but does not preserve the gene-gene correlations.

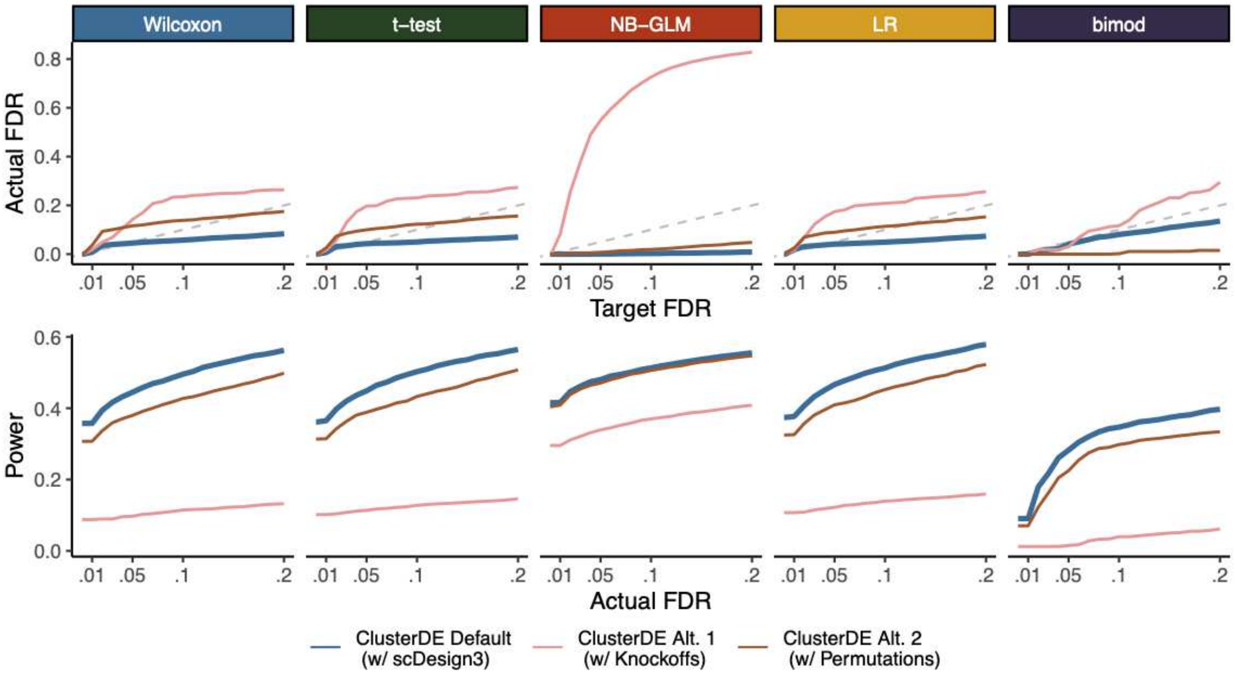

Figure S8: The FDRs and power of ClusterDE with three approaches for synthetic null generation: scDesign3 (the default in ClusterDE), the model-X knockoffs, and independent permutations of all genes across cells. Compared with the other two approaches, scDesign3 controls the FDR and yields higher power.

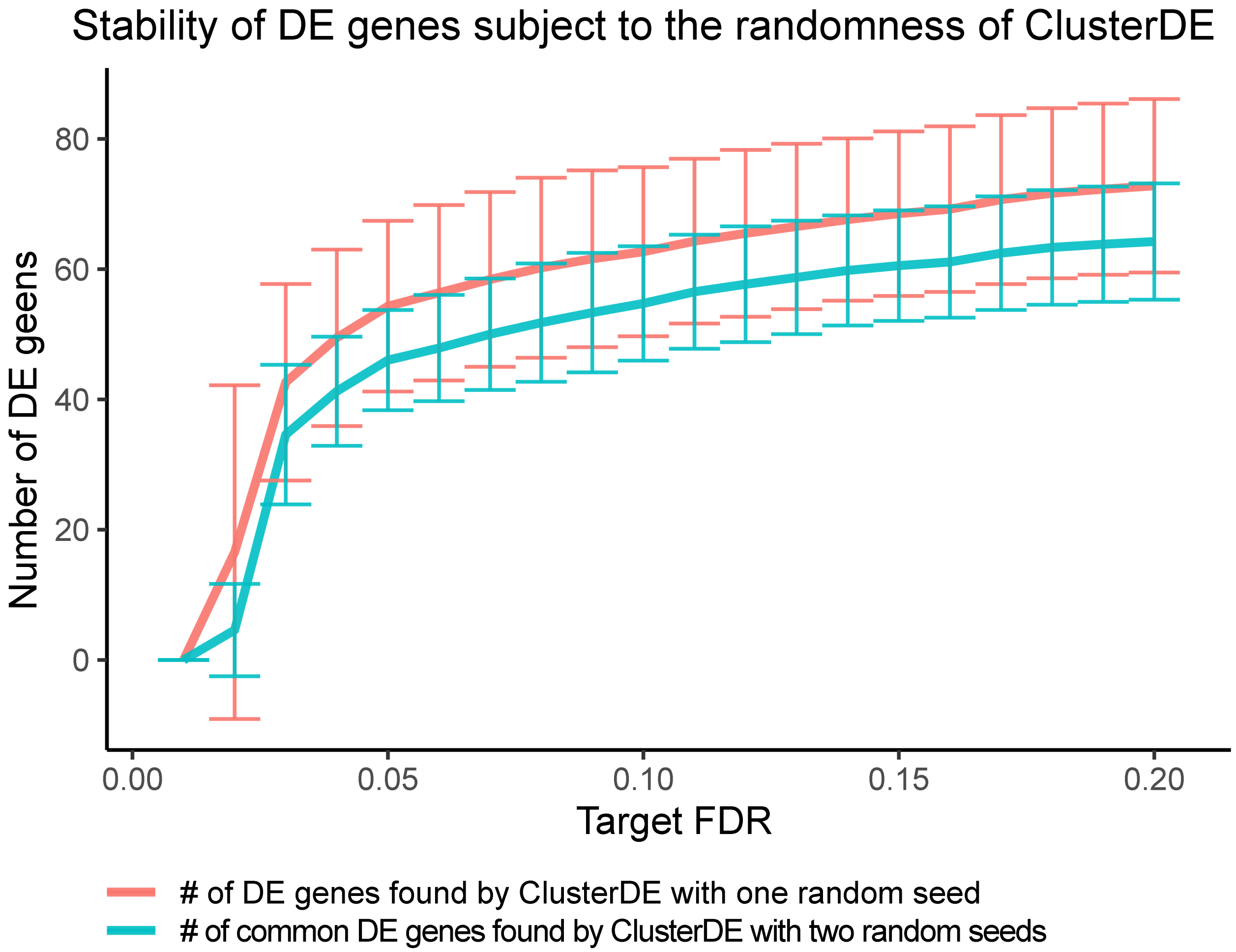

Figure S9: Stability of the DE genes identified by Cluster in relation to the randomness of synthetic null generation. Given one target dataset simulated with two cell types (see **Methods** "Simulation design with two cell types and 200 DE genes"), 50 synthetic null datasets are generated with 50 random seeds, and DE genes are identified by ClusterDEusing each syntheticnull dataset. The red curve shows the mean and standard deviation (half of the vertical bar height) of the numbers of DE genes identified at each target FDR across the 50 random seeds. The cyan curve shows the mean and standard deviation (half of the vertical bar height) of the numbers of DE genes shared between two random seeds, across $\binom{50}{2}$ pairs of random seeds, at each target FDR. The results show that the DE genes identified by ClusterDE remain relatively stable and robust to the randomness.

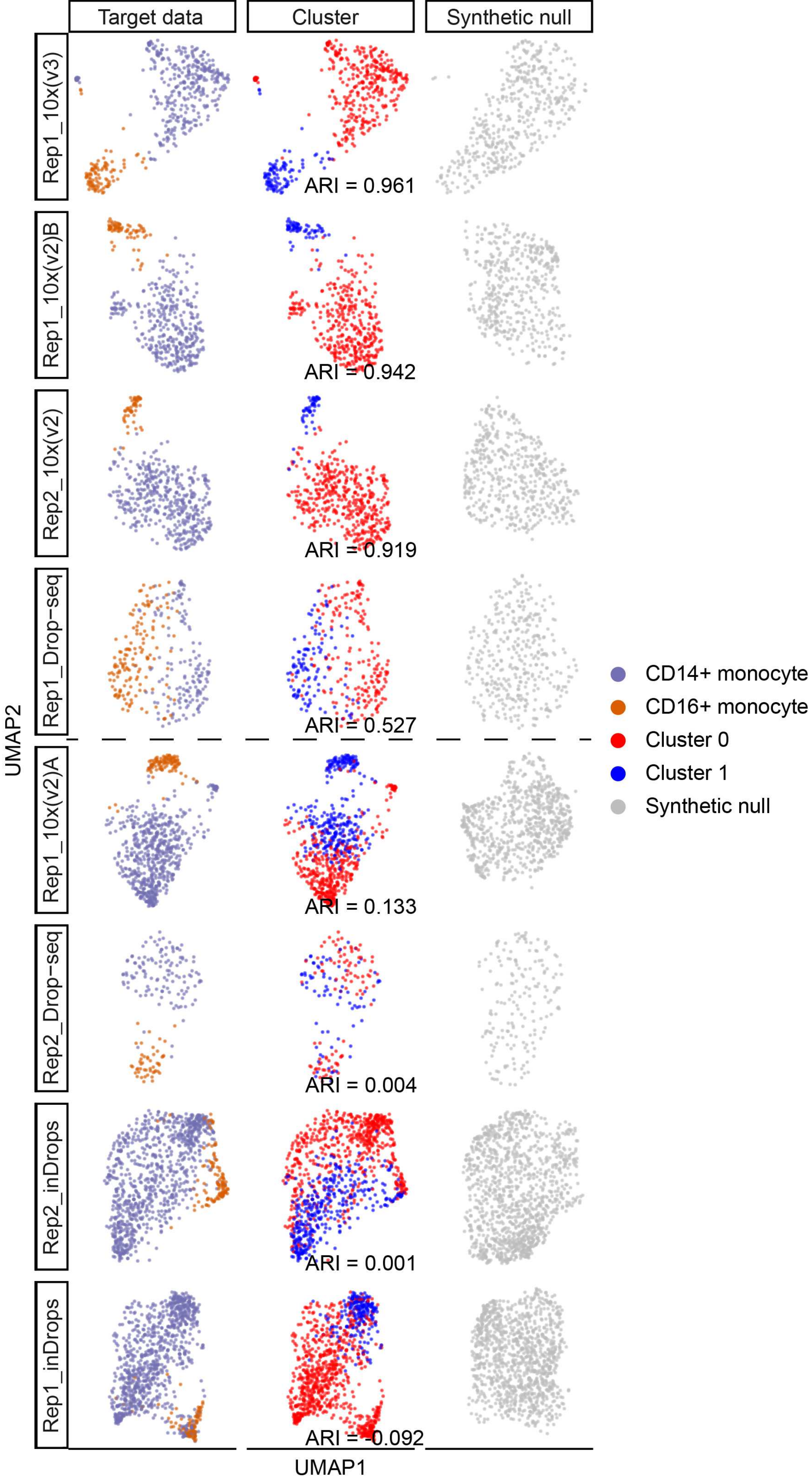

Figure S10: UMAP visualizations and Seurat clustering accuracies (ARIs) of the eight PBMC monocyte datasets (ordered by ARIs from high to low). The first and second columns show the UMAP visualizations of the eight datasets as the target data, with the cells labeled by the monocyte subtypes (the first column) or the clusters (the second column). The third column shows the UMAP visualizations of the synthetic null data corresponding to the eight target datasets. The horizontal dashed line between rows 4 and 5 divides the eight datasets based on the clustering accuracy. It is expected that monocyte-subtype markers are more likely to be identified as post-clustering DE genes from the top four datasets than the bottom four datasets.

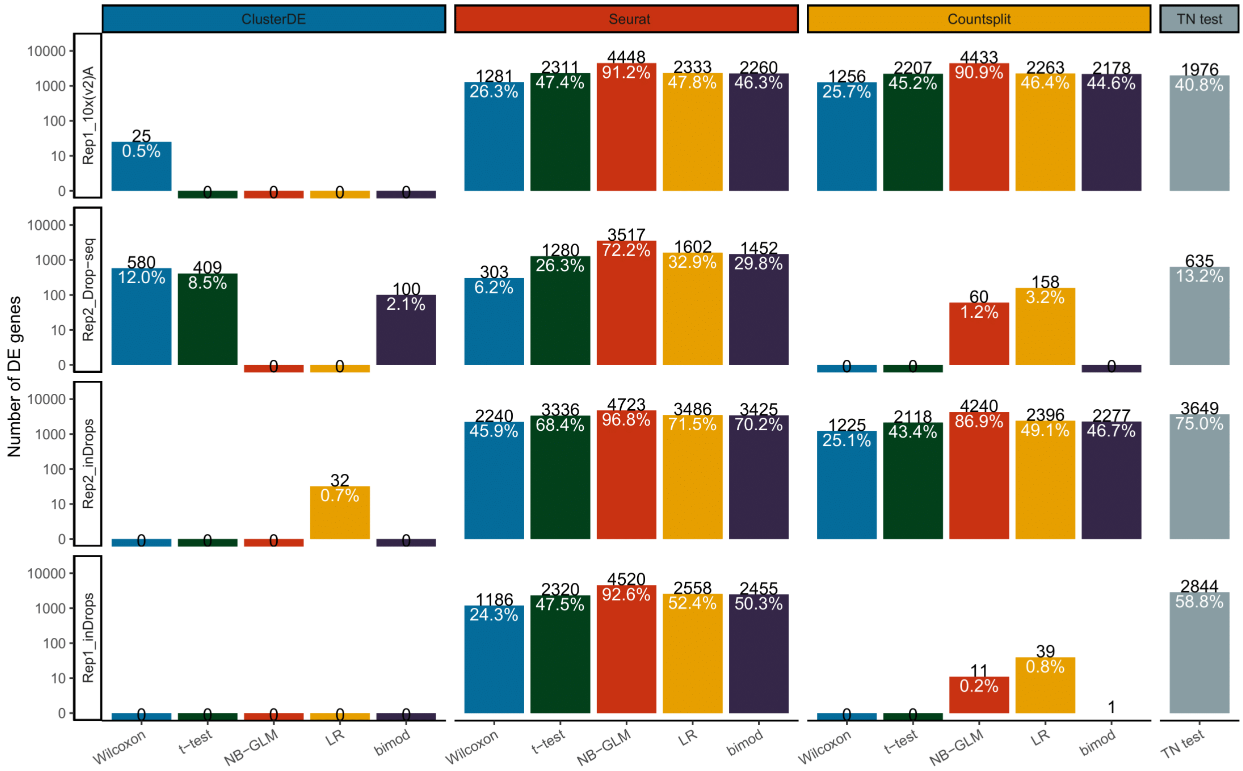

Figure S11: ClusterDE avoids false discoveries under double dipping. Although the datasets contain two monocyte subtypes, the clustering results poorly match the subtype labels (the bottom four datasets in Fig. S10), and thus no DE genes should be discovered. The numbers in black and white are the number of DE genes and the proportion of DE genes among all genes, respectively. In most cases, ClusterDE does not find DE genes, as expected.

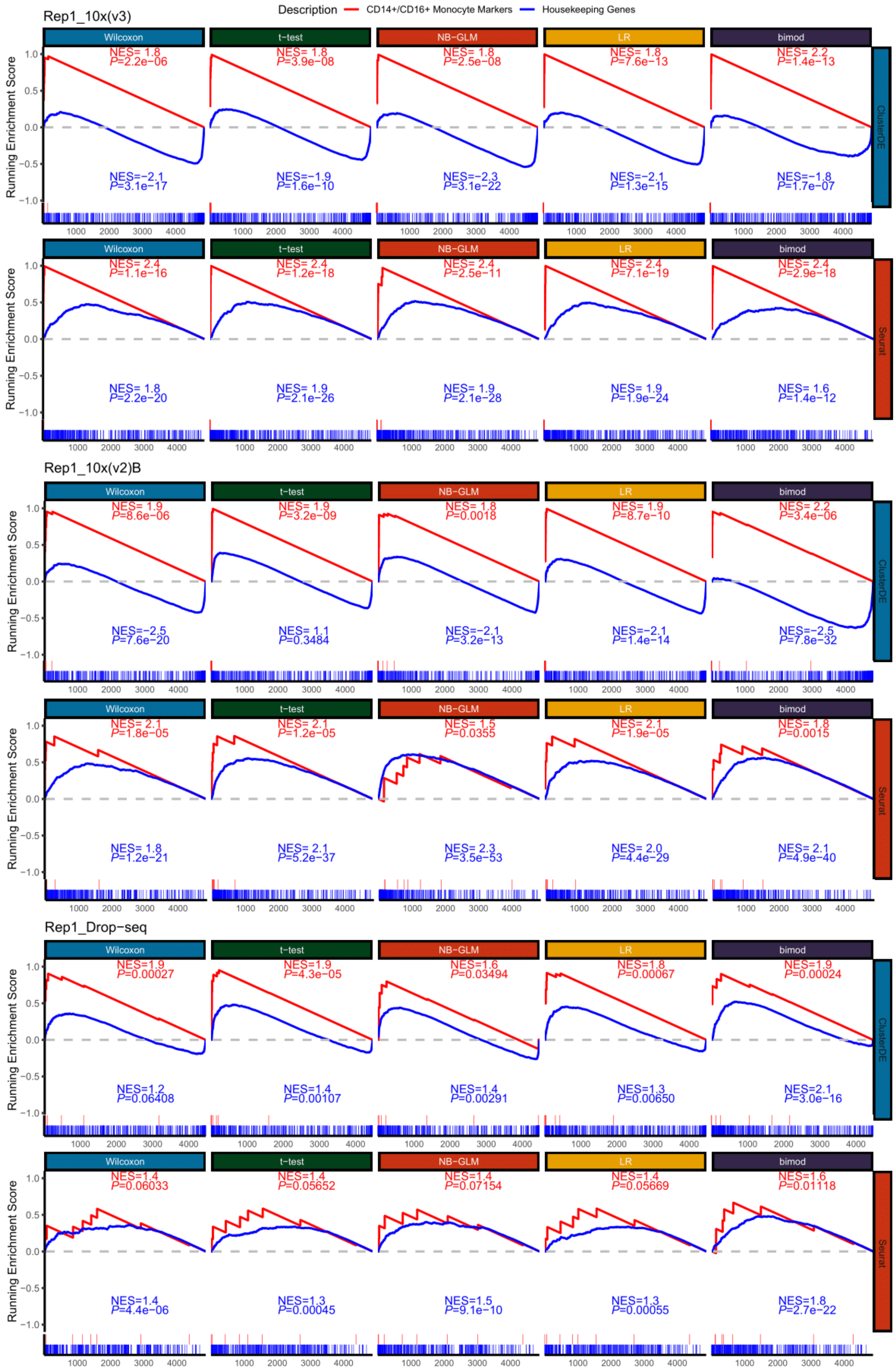

Figure S12: Gene set enrichment analysis (GSEA) of the ranked DE gene lists identified by ClusterDE and Seurat with five DE tests from three datasets. The red lines represent the enrichment of the "CD14$^+$/CD16$^+$ Monocyte Marker Genes" set, and the blue lines represent the enrichment of the "Housekeeping Genes" set. The short vertical lines at the bottom show the rank distributions of the genes in the two gene sets within each ranked DE gene list. The normalized enrichment score (NES) reflects the direction and magnitude of enrichment, and the P value indicates the significance of enrichment.

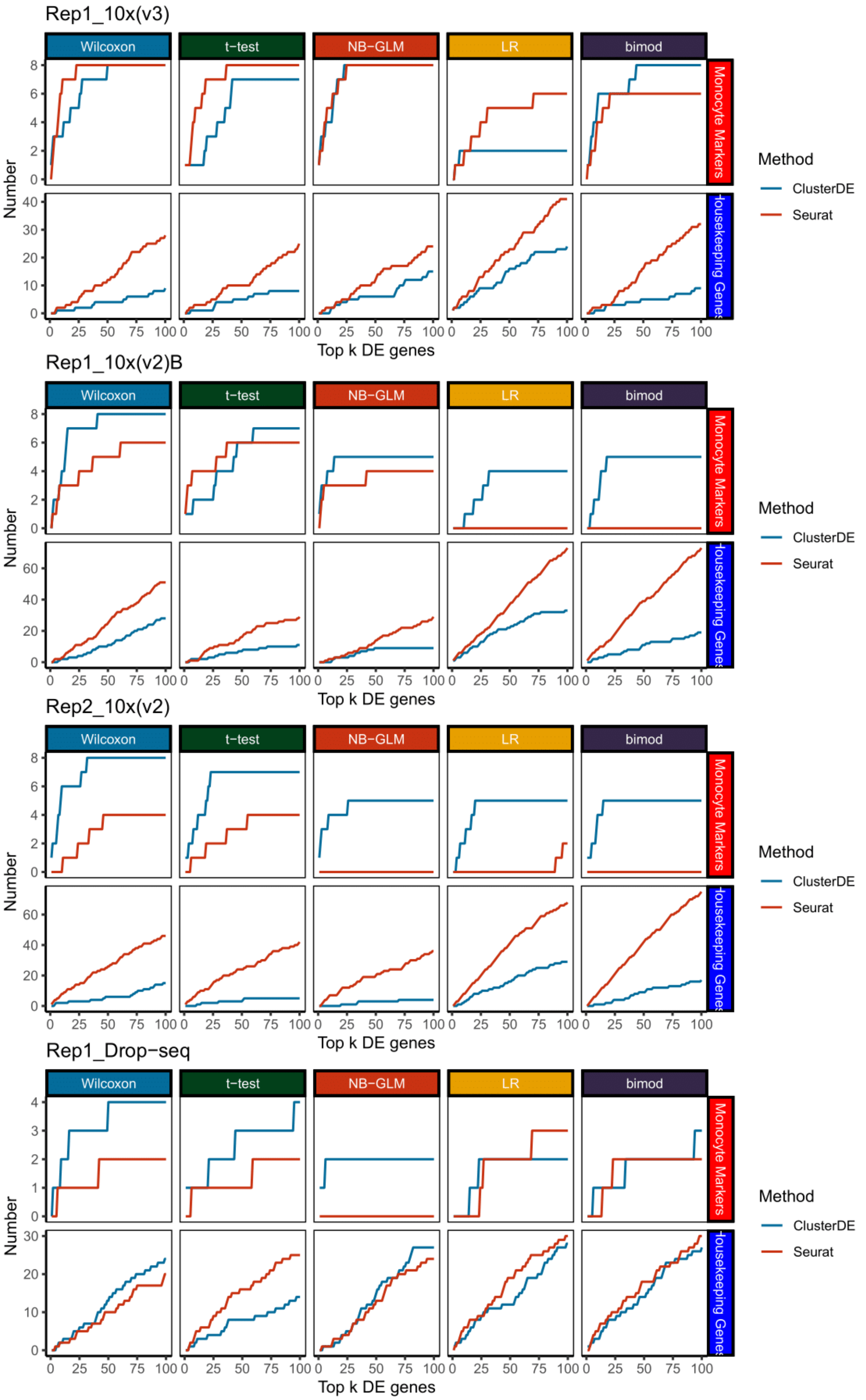

Figure S13: Overlaps between monocyte markers/housekeeping genes and the top k DE genes, with k ranging from 1 to 100. The horiontal axis represents k (for example, $k = 100$ means we select the top 100 DE genes from the DE gene lists). The vertical axis indicates the number of monocyte markers (the top row in each panel) or housekeeping genes (the bottom row in each panel) among the top k DE genes found by each of the five DE tests (columns). In most cases, ClusterDE (blue line) identifies more monocyte subtype markers and fewer housekeeping genes compared to Seurat (red line).

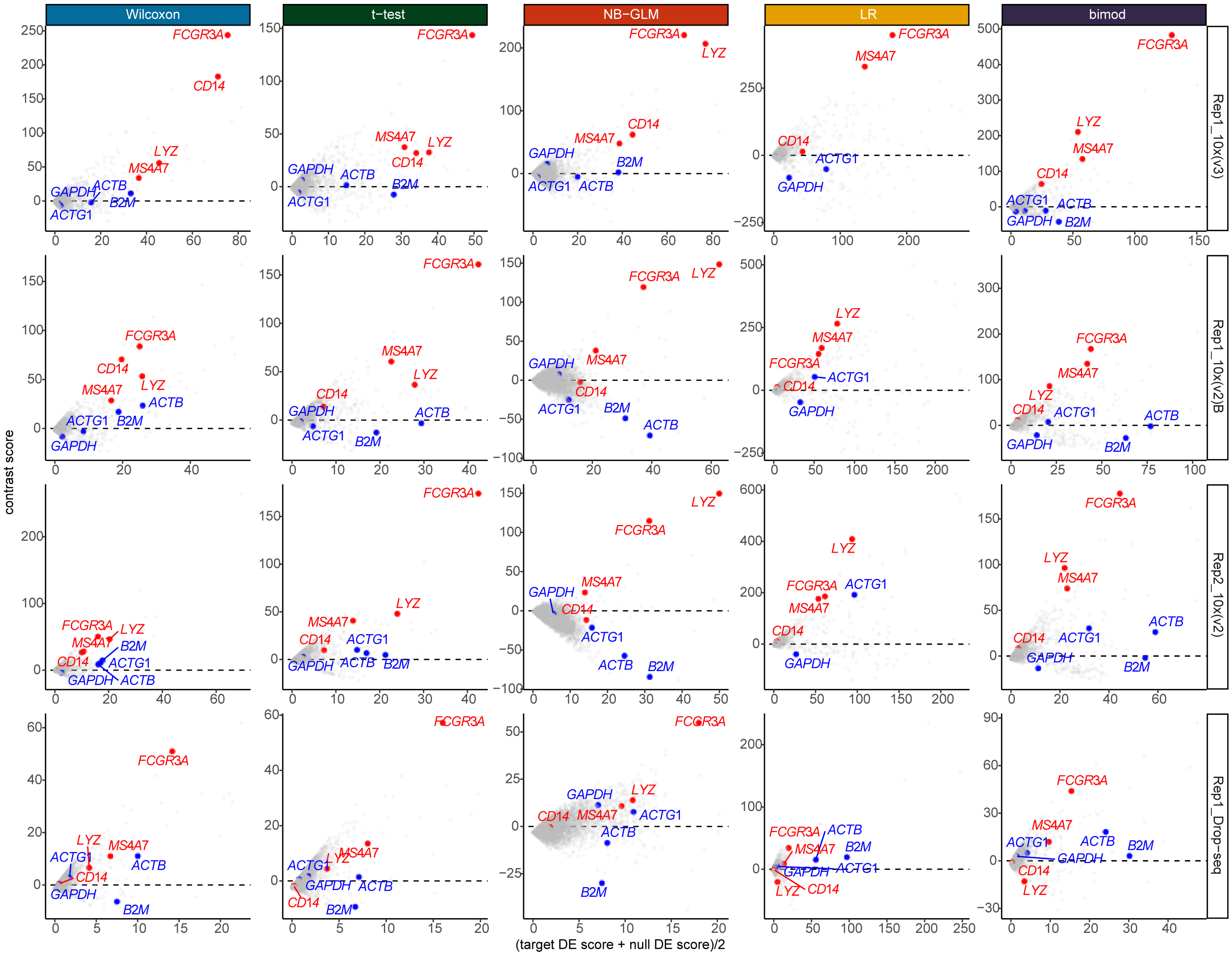

Figure S14: The minus-average (MA) plots of ClusterDE contrast scores (target DE score minus null DE score) vs. averages of target DE scores and null DE scores. The red color labels four well-known CD14$^+$/CD16$^+$ subtype markers, and the blue color labels four well-known housekeeping genes. The dashed black line indicates the contrast scores of 0. For housekeeping genes, their DE scores are large in both target data and synthetic null data, so their contrast scores are centered around 0. Hence, these housekeeping genes would be ranked top by Seurat (which only examines target DE scores) but not by ClusterDE. On the other hand, the DE scores of subtype markers are much larger in target data than in synthetic null data, so their contrast scores are large and ranked top by ClusterDE.

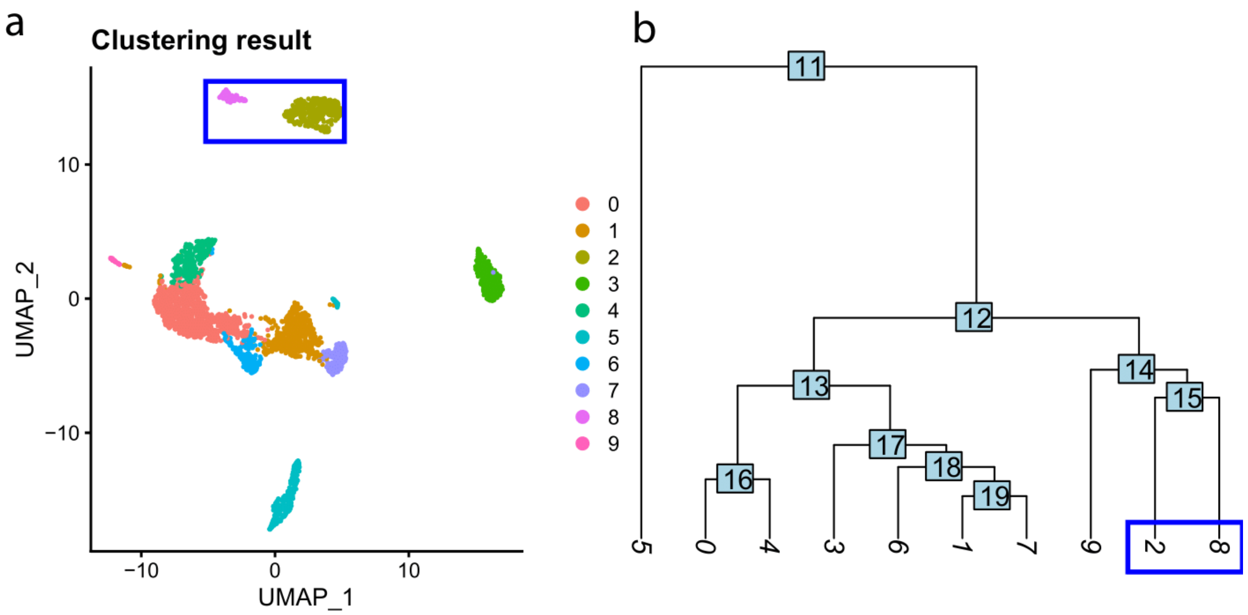

Figure S15: A demonstration of using ClusterDE in the presence of multiple cell clusters. **a**, A UMAP visualization of Seurat clusters found in the PBMC dataset Rep1 10x(v3). The blue box labels two neighboring clusters, which roughly represent the $CD14^{+}/CD16^{+}$ monocytes. **b**, The cluster tree constructed by Seurat, with clusters 2 and 8 corresponding to the two clusters in the blue box in **a**. It is recommended that ClusterDE can help annotate two neighboring cell clusters in UMAP or a cluster tree, based on the more trustworthy post-clustering DE genes identified by ClusterDE. The reason is that neighboring clusters are more likely to be spurious.

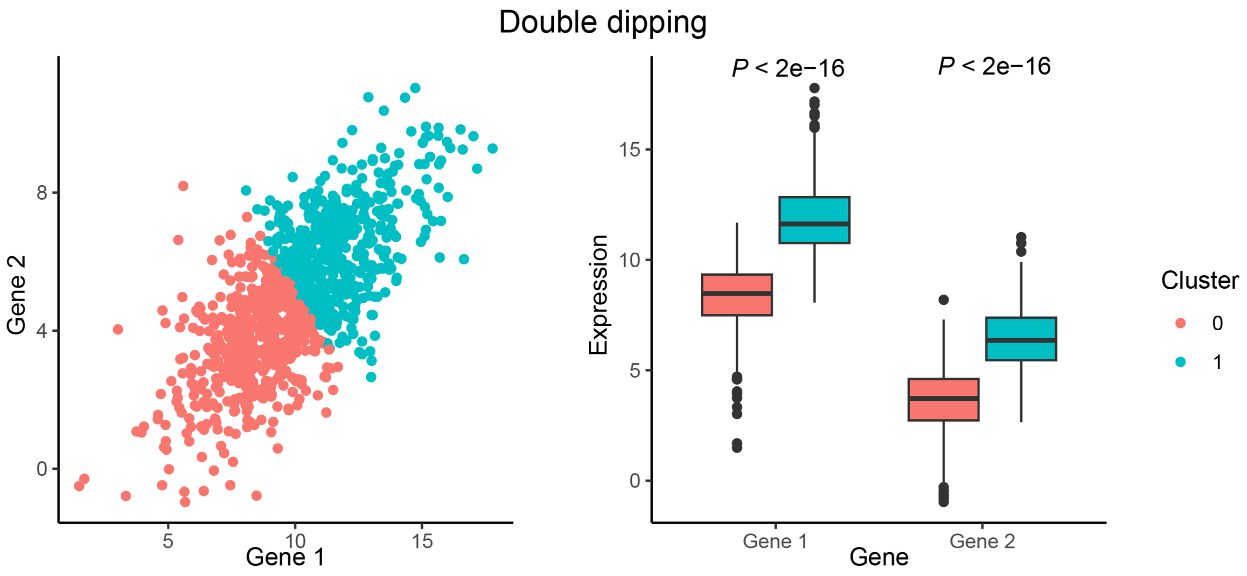

Figure S16: A toy example to showcase the double-dipping issue. The two genes' expressions follow a bivariate Gaussian distribution as the cells come from a homogeneous cell type. However, if we run the K-means clustering to divide the cells into two clusters, the two genes are forced to exhibit different distributions in the two clusters.
